# Risk Factors for Severe Bronchiolitis in Australian and Aotearoa New Zealand Infants: A Systematic Review

**DOI:** 10.1111/jpc.70165

**Published:** 2025-08-08

**Authors:** Kate Loveys, Meredith L. Borland, Ed Oakley, Franz E. Babl, Elizabeth Cotterell, Libby Haskell, Sharon O’Brien, Catherine L. Wilson, Emma J. Tavender, Stuart R. Dalziel, the Paediatric Research in Emergency Departments International Collaborative (PREDICT) Network

**Affiliations:** ^1^ Department of Paediatrics: Child and Youth Health, School of Medicine The University of Auckland Auckland New Zealand; ^2^ Emergency Department Perth Children’s Hospital Perth Western Australia Australia; ^3^ Divisions of Emergency Medicine and Paediatrics The University of Western Australia Perth Western Australia Australia; ^4^ Department of Paediatrics The University of Melbourne Melbourne Victoria Australia; ^5^ Department of Critical Care The University of Melbourne Melbourne Victoria Australia; ^6^ Emergency Department Royal Children’s Hospital Melbourne Victoria Australia; ^7^ Emergency Research, Clinical Sciences Murdoch Children’s Research Institute Melbourne Victoria Australia; ^8^ Armidale Rural Referral Hospital Armidale New South Wales Australia; ^9^ School of Rural Medicine and Tablelands Clinical School, University of New England Armidale New South Wales Australia; ^10^ Emergency Department Starship Children’s Hospital Auckland New Zealand; ^11^ Institute for Paediatric Perioperative Excellence, The University of Western Australia Perth Western Australia Australia; ^12^ Australian Catholic University Sydney New South Wales Australia; ^13^ Department of Surgery, School of Medicine The University of Auckland Auckland New Zealand

**Keywords:** Australia, bronchiolitis, infant, New Zealand, risk factors, systematic review

## Abstract

**Aim:**

Bronchiolitis is the leading cause of hospital admission in Australasian infants. Infants with risk factors for severe disease may have a greater likelihood of prolonged hospitalisation and intensive care admission. This study aimed to synthesise the literature on risk factors for severe bronchiolitis in Australasian infants.

**Methods:**

Systematic review including observational studies of risk factors for severe bronchiolitis in Australasian infants (< 12 months), published from 2000. Databases were searched (24 January 2024): MEDLINE, EMBASE, PubMed, Cochrane Library and CINAHL. Risk of bias (RoB) was assessed using the Newcastle–Ottawa Scale for cohort studies, and evidence quality was evaluated using GRADE. Results were narratively synthesised.

**Results:**

Ten out of 26 467 articles were included (*N* = 895 276; 12 cohorts, prospective = 5, retrospective = 7). Studies were mostly rated low RoB. There was evidence for the following risk factors: younger chronological age, prematurity, plural birth, comorbidity (chronic lung disease, congenital heart disease, chronic neurological disease, any genetic disorder, any comorbidity), Indigenous ethnicity, economic disadvantage, tobacco smoke exposure and timing of illness onset at presentation. Most risk factors had moderate‐quality evidence (range high to very low). Evidence was lacking for the following risk factors present in international literature: breastfeeding exposure and faltering growth. The following risk factors have not been reported in Australasian infants: trisomy‐21, congenital diaphragmatic hernia and environmental pollutants.

**Conclusions:**

Risk factors for severe bronchiolitis in Australasian infants are largely consistent with the international literature, although evidence is lacking for some. Knowledge of these risk factors is highly relevant to those assessing infants with bronchiolitis, and in guiding targeted delivery of respiratory syncytial virus immunisation and other preventative programmes.

**Trial Registration:**

PROSPERO (CRD42023463917)

**Summary**

- A systematic review was conducted to synthesise observational evidence on risk factors for severe bronchiolitis in Australasian infants.
- Based on moderate‐quality evidence from five prospective and seven retrospective cohort studies in Australasian infants (*N* = 895 276), the following risk factors for severe bronchiolitis were identified: younger chronological age, prematurity (< 37 weeks gestational age), plural birth, comorbidity (chronic lung disease, congenital heart disease, chronic neurological disease, any genetic disorder, any comorbidity), Indigenous ethnicity, economic disadvantage, tobacco smoke exposure and time of illness onset at presentation.
- Evidence was not found for the following risk factors present in the international literature: breastfeeding exposure and faltering growth.
- The following risk factors have not been specifically reported in Australasian infants: trisomy 21, congenital diaphragmatic hernia and environmental pollutants. However, they are likely applicable.
- Clinicians should take these risk factors into account when assessing and managing infants with bronchiolitis in Australasian hospitals. The risk factors may additionally inform the targeted delivery of respiratory syncytial virus (RSV) immunisation and other preventative programmes in Australasian infants.

## 1 Introduction

Bronchiolitis is the largest cause of hospital admission for infants in Australia and Aotearoa New Zealand (AoNZ) [1, 2]. Bronchiolitis is an acute respiratory infection in infants (< 12 months of age) that is triggered by a viral infection, most often with respiratory syncytial virus (RSV) [3]. It is characterised by signs of an upper respiratory tract infection (e.g., rhinorrhoea, nasal congestion and cough), followed by signs of a lower respiratory tract infection, including respiratory distress and the presence of diffuse crackles or wheeze.

Most bronchiolitis is self‐resolving; however, a proportion of infants develop a more severe illness requiring prolonged hospitalisation and intensive care unit (ICU) level care [3]. It is important that clinicians identify the presence of risk factors for severe illness at hospital presentation to inform patient management, such as the need for monitoring, admission, earlier use of interventions, and escalated care. Identification of risk factors may also inform targeted delivery of preventative interventions, such as monoclonal antibody vaccines for RSV [4, 5] or other respiratory viruses that may cause bronchiolitis (e.g., human metapneumovirus [hMPV] [6]).

As part of the 2016 Australasian Bronchiolitis Guideline [7], the Paediatric Research in Emergency Departments International Collaborative (PREDICT) recommended that clinicians consider several risk factors for severe illness when managing infants with bronchiolitis, including gestational age under 37 weeks, chronological age at presentation under 10 weeks, exposure to cigarette smoke, breastfeeding under 2 months, failure to thrive, Indigenous ethnicity, and the presence of chronic lung disease, heart and/or neurological conditions (recommendation strength: *conditional*; evidence quality: *low*).

Although this recommendation was based on the results of 22 observational studies [8, 9, 10, 11, 12, 13, 14, 15, 16, 17, 18, 19, 20, 21, 22, 23, 24, 25, 26, 27, 28, 29, 30, 31], these were mostly international data rated as low‐quality, with a lack of Australasian‐specific data. To date, other systematic reviews have concentrated on single risk factors for severe bronchiolitis (e.g., congenital heart disease [32]) or have focused on risk factors for severe bronchiolitis in specific regions (e.g., the United States [33] and Europe [34]). This systematic review aimed to synthesise the literature on risk factors for severe bronchiolitis in Australian and AoNZ infants, and place it within an international context.

## 2 Materials and Methods

A systematic review, prospectively registered on PROSPERO (CRD42023463917; details protocol), was conducted as part of the Australasian Bronchiolitis Guideline 2025 project [35]. There were no major deviations from the pre‐registered methodology. The Preferred Reporting Items for Systematic Reviews and Meta‐Analyses (PRISMA) 2020 guidance was followed (Appendix S1) [36].

### 2.1 Search Strategy

Systematic searches were performed by a subject librarian on electronic databases, Ovid MEDLINE, Ovid EMBASE, PubMed, CINAHL and the Cochrane Library (last search 24 January 2024) (details in Appendices S2 and S3). Manual searches were conducted from conference abstracts and reference lists of prior reviews. Subject matter experts in the review team assessed the included article list to ensure no key studies were missing.

### 2.2 Study Selection

Study selection was performed by all authors using Covidence software (Veritas Health Innovation, Melbourne, Australia), independently and in duplicate, in two stages: title and abstract, full‐text screening. Training took place prior to screening. Rating disputes were resolved through consensus involving ≥ 1 additional researcher. We included peer‐reviewed journal articles of observational studies that reported on risk factors for severe bronchiolitis in infants (< 12 months of age) located in Australia and/or AoNZ, published in English from 2000 onwards. Table 1 presents eligibility criteria.

**TABLE 1:** Eligibility criteria.

	Detailed criteria
Inclusion criteria	Population: Infants < 12 months of age with bronchiolitis. As bronchiolitis is commonly caused by a respiratory syncytial virus (RSV) infection, studies in infants with confirmed RSV infection were eligible. Independent variable: Risk factors for severe bronchiolitis. Comparator: No criteria. Outcomes: To be included, the study needed to report on the statistical association between any risk factor and at least one of the following illness severity outcomes: ICU admission, death, mechanical ventilation, hospital admission, length of stay, time on positive pressure ventilation support (high flow therapy, continuous positive airway pressure, mechanical ventilation). We required studies to report adjusted or unadjusted risk ratios (RRs), odds ratios (ORs), hazard ratios (HRs) or incidence rate ratios (IRRs) for dichotomous data, or mean difference (MD) or median difference values for continuous data. Setting: Population based in Australia or Aotearoa New Zealand. Study design: Studies of any observational research design were included. Publication characteristics: Peer‐reviewed journal articles published in the English language from 2000 onwards were included.
Exclusion criteria	Studies in mixed populations where < 75% of the sample were infants < 12 months of age diagnosed with bronchiolitis or RSV, if the data were not separately reported for this group. Studies reporting on clinical signs (e.g., vitamin D levels), as opposed to risk factors. Publication type that was not a peer‐reviewed journal article. For example, conference abstracts, reports and theses. Publications not reporting the results of a primary research study. For example, reviews, viewpoints or letters to the editor. International studies without data separately reported for Australian and/or AoNZ infants.

### 2.3 Data Extraction

Data were extracted into a custom spreadsheet. One researcher extracted the data, with review by a second; no automation tools were used. No data were required to be sought from the study authors. Appendix S3 presents the data items extracted. All results for an outcome were extracted, irrespective of the measure or time point, provided the statistical criteria were met (Table 1).

### 2.4 Risk of Bias (RoB) Assessment

RoB of included studies was assessed using the Newcastle–Ottawa Scale for cohort studies (see Appendix S3 for details) [37]. Assessments were performed by one researcher, with review by a second. A higher score (≥ 7) indicates lower RoB.

### 2.5 Data Synthesis

A narrative synthesis was performed [38] as quantitative synthesis was not possible due to the small number of studies per risk factor, which often did not overlap in terms of outcomes, or were too heterogeneous in design or analytical strategy (e.g., adjustments for covariates). There were too few studies per risk factor (< 10) to assess publication bias. There were no missing data or data conversions required. The effect estimates (OR, RR, HR, IRR, MD and median difference) and 95% confidence intervals (CIs) were tabulated by risk factor. The narrative summary discusses possible sources of heterogeneity. Risk factors identified from included studies were compared to those identified in systematic reviews of the international literature [39, 40, 41, 42, 43, 44, 45, 46, 47, 48, 49].

### 2.6 Certainty of Evidence Assessment

The evidence quality per risk factor was evaluated using the Grading of Recommendations, Assessment, Development, and Evaluation (GRADE) methodology [50]. The appraisals were made by one researcher with review by a second. The quality of the evidence was assessed by outcome within a topic across domains of RoB, inconsistency, indirectness, imprecision, and other factors (e.g., publication bias). GRADEpro GDT software was used [51].

## 3 Results

### 3.1 Literature Search

**FIGURE 1:**
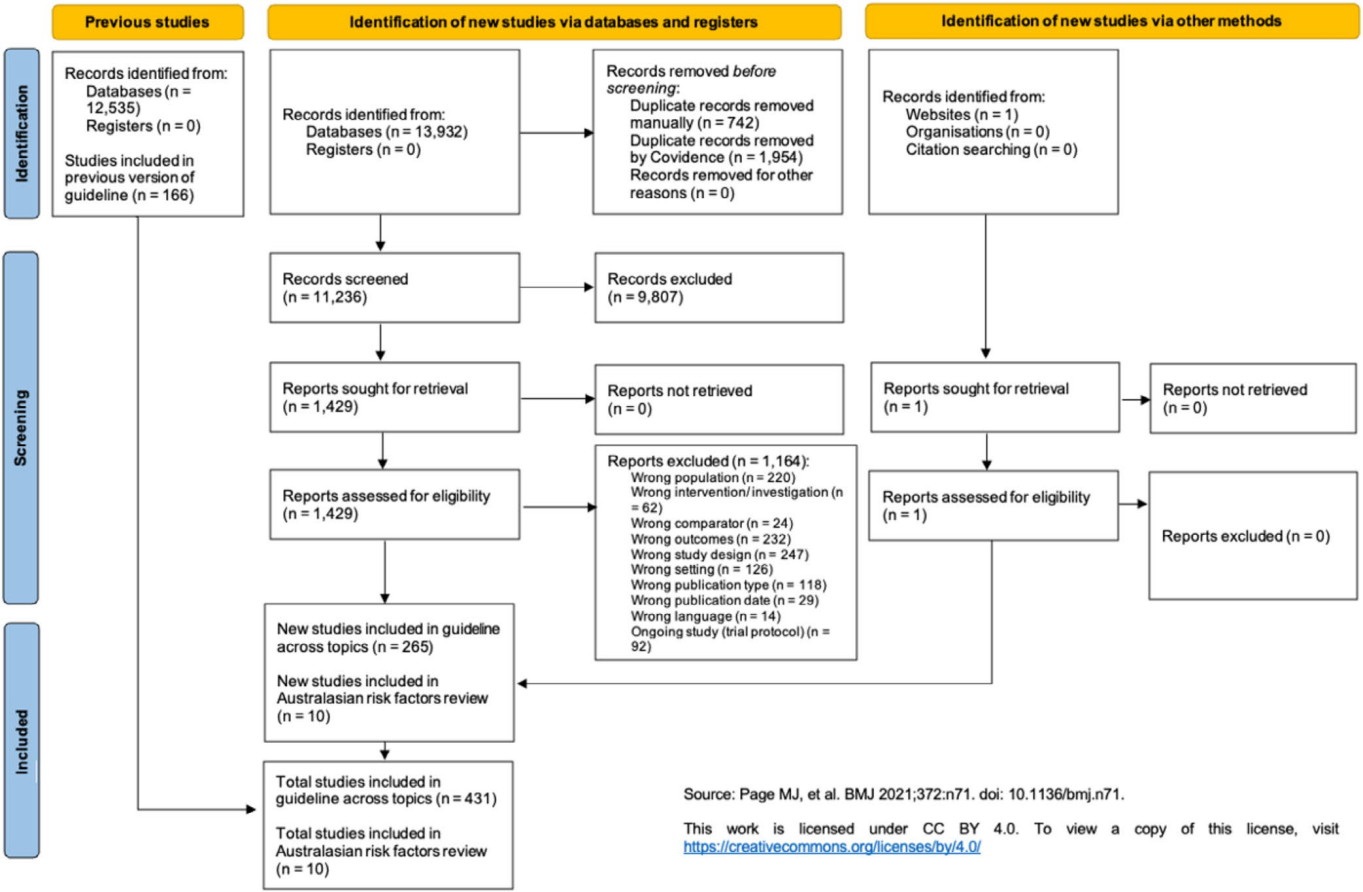
PRISMA flowchart.

Ten studies were included from 26 467 records identified for the 2016 (*n* = 12 535) and 2025 Australasian Bronchiolitis Guidelines (*n* = 13 932) (Figure 1). Related but ineligible studies were excluded for not meeting the statistical reporting criteria [52, 53, 54, 55] or reporting on non‐Australasian populations [56, 57, 58].

Study characteristics are presented in Appendix S4. We included five prospective and seven retrospective cohort studies, conducted in Australia (*n* = 6), AoNZ (*n* = 1) or both countries (*n* = 3). Data were collected between 2001 and 2016. Overall, there were 895 276 participants (range: 282–866 262). Participant mean age ranged from 3.69 to 6.40 months (unreported in four studies). The proportion of Indigenous participants per study ranged from 3% to 100% (unreported in four studies).

### 3.2 RoB

RoB appraisals are presented in Table 2. All studies received a total score of ≥ 7 (low RoB). Studies were downgraded for comparability of the cohorts (*n* = 5 studies) and for insufficient demonstration that the outcome was not present at study initiation (*n* = 3 studies). All studies were allocated maximum stars for the remaining domains.

**TABLE 2:** Summary of risk of bias (RoB) from the Newcastle–Ottawa scale for cohort studies [37].

Study ID	Selection domain				Comparability domain	Outcome domain			Total score
	Representativeness of the exposed cohort^a^	Selection of the non‐exposed cohort^a^	Ascertainment of exposure^a^	Demonstration of the outcome of interest not present at study start^a^	Comparability of cohorts on basis of design or analysis^b^	Outcome assessment^a^	Adequate length of follow‐up^a^	Adequacy of follow‐up of cohorts^a^	
Butler et al. 2019	*	*	*	—	**	*	*	*	8
Franklin et al. 2023	*	*	*	*	**	*	*	*	9
Homaira et al. 2016	*	*	*	*	**	*	*	*	9
McCallum et al. 2016	*	*	*	*	*	*	*	*	8
Oakley et al. 2017	*	*	*	*	—	*	*	*	7
Pham et al. 2020	*	*	*	*	**	*	*	*	9
Prasad et al. 2020	*	*	*	*	—	*	*	*	7
Saravanos et al. 2019	*	*	*	—	*	*	*	*	7
Schlapbach et al. 2017	*	*	*	—	**	*	*	*	8
Stevenson et al. 2023	*	*	*	*	*	*	*	*	8

^a^ Maximum 1 star score.

^b^ Maximum 2 star score.

### 3.3 Risk Factors for Severe Bronchiolitis in Australasian Infants

The risk factors investigated and a summary of their effect estimates are presented in Table 3. Some studies were conducted in subgroups, including ICU patients, Neonatal ICU (NICU) graduates, hypoxaemic infants and Indigenous infants, which may have contributed to the variation in effect estimates.

**TABLE 3:** Summary of the evidence for risk factors of severe bronchiolitis in Australasian infants.

Risk factor	Severity outcome					
	Effect estimate (95% CI)					
	ICU admission	Death	Mechanical ventilation	Hospital admission	Length of stay	Time on PPVS
Younger chronological age	Adj OR 0.98 (0.96–0.99)*** [59] Age in weeks Hypoxaemic infants	—	Adj OR 0.97 (0.96–0.98)*** [60] Age days/30 ICU patient subgroup	Adj OR 0.95 (0.90–0.99)* [61] Age in months Severe RSV subgroup	Median difference −0.6 h (−1.6 to 0.5) [62] Age in months Indigenous subgroup	—
	OR 1.5 (1.1–2.0)** ^a^ [63] Age 2 to < 6 m vs. ≥ 6 m	—	—	IRR 1.9 (1.8–2.0)* ^a^ [64] Age < 6 m Indigenous vs. non‐Indigenous	—	—
	—	—	—	IRR 1.6 (1.5–1.7)* ^a^ [64] Age 0–2 m Indigenous vs. non‐Indigenous	—	—
	—	—	—	IRR 2.5 (2.3–2.6)* ^a^ [64] Age 3–5 m Indigenous vs. non‐Indigenous	—	—
	—	—	—	IRR 2.1 (2.0–2.3)* ^a^ [64] Age in months Indigenous vs. non‐Indigenous	—	—
	—	—	—	IRR 0.64 (0.59–0.69)*** ^a^ [65] Age 6–11 m vs. 0–5 m NICU graduates subgroup	—	—
	—	—	—	RR 2.54 (2.10–3.06)* ^a^ [66] Age < 3 m vs. 6–11 m	—	—
Prematurity	Adj OR 1.29 (0.77–2.14) [59] < 37wGA Hypoxaemic subgroup	—	Adj OR 1.32 (1.15–1.52)*** [60] < 37wGA ICU patient subgroup	IRR 6.78 (6.07–7.57)*** ^a^ [65] < 28wGA NICU graduate subgroup	Median difference 0.7 h (−0.7 to 2.0)^a^ [62] wGA Indigenous subgroup	—
	OR 1.5 (1.0–2.1)* ^a^ [63] < 37wGA	—	—	IRR 3.30 (2.98–3.64)*** ^a^ [65] 28–32wGA NICU graduate subgroup	—	—
	—	—	—	IRR 1.56 (1.44–1.70)*** ^a^ [65] 33–36wGA NICU graduate subgroup	—	—
	—	—	—	OR 2.5 (1.3–5.0)** ^a^ [61] < 33wGA Severe RSV subgroup	—	—
	—	—	—	OR 1.5 (0.66–3.6)^a^ [61] 33–34 + 6wGA Severe RSV subgroup	—	—
	—	—	—	OR 0.87 (0.35–2.2)^a^ [61] 35–37wGA Severe RSV subgroup	—	—
Plural birth	—	—	—	Adj HR 1.20 (1.07–1.35)* [67] NIHR subgroup	—	—
	—	—	—	Adj HR 1.47 (1.28–1.70)* [67] NISR subgroup	—	—
	—	—	—	HR 1.05 (0.64–1.73)^a^ [67] Indigenous subgroup	—	—
Faltering growth/slow weight gain (failure to thrive)	Adj OR 0.99 (0.84–1.18) [59] Admission weight (kg) Hypoxaemic subgroup	—	—	—	Median difference −0.0 h (−5.7 to 6.2)^a^ [62] Birth weight (kg) Indigenous subgroup	—
Chronic lung disease/bronchopulmonary dysplasia	OR 1.6 (1.0–2.6)* ^a^ [63]	—	Adj OR 1.59 (1.18–2.12)** [60] ICU subgroup	IRR 5.12 (4.64–5.65)*** ^a^ [65] Age 6–11 m vs. 0–5 m NICU graduates subgroup	—	—
	—	—	Adj OR 1.69 (1.32–2.15)*** [60] ICU subgroup BPD	Adj OR 2.6 (1.4–4.9)* [61] Severe RSV subgroup	—	—
Congenital heart disease (CHD)	OR 2.3 (1.5–3.5)*** ^a^ [63]	—	Adj OR 1.88 (1.54–2.29)*** [60] ICU subgroup	Adj OR 2.7 (1.1–6.4)* [61] Severe RSV subgroup	—	—
Chronic neurological condition	OR 2.2 (1.2–4.1)** ^a^ [63]	—	Adj OR 1.72 (1.19–2.50)** [60] ICU subgroup	—	—	—
Any genetic disorder	—	—	—	OR 4.2 (1.0–17.0)* ^a^ [61] Severe RSV subgroup	—	—
Any comorbidity	—	Adj OR 2.59 (1.42–4.70)* [68] ICU subgroup	Adj OR 1.97 (1.39–2.79)*** [68] ICU subgroup	—	—	Median difference 26.8 h longer (95% CI NR)^a^ [68] ICU subgroup
Low breastfeeding exposure	—	—	—	—	Median difference 1.9 h (−8.9 to 8.0)^a^ [62] Indigenous subgroup Currently breastfed vs. not	—
Tobacco smoke exposure	—	—	—	Adj HR 1.39 (1.20–1.61)* [67] Indigenous subgroup	Median difference 1.7 h (−6.6 to 11.4)^a^ [62] Indigenous subgroup Prenatal exposure	—
	—	—	—	Adj HR 1.26 (1.13–1.41)* [67] NIHR subgroup	Median difference 0.2 h (−8.0 to 9.8)^a^ [62] Indigenous subgroup Postnatal household exposure	—
	—	—	—	Adj HR 1.47 (1.40–1.55)* [67] NISR subgroup	—	—
Indigenous ethnicity	—	—	—	IRR 1.6 (1.5–1.7)* ^a^ [64] Age 0–2 m	—	—
	—	—	—	IRR 2.5 (2.3–2.6)* ^a^ [64] Age 3–5 m	—	—
	—	—	—	IRR 1.9 (1.8–2.0)* ^a^ [64] Age < 6 m	—	—
	—	—	—	IRR 2.1 (2.0–2.3)* ^a^ [64] Age 6–11 m	—	—
	—	—	—	Adj OR 2.6 (1.4–4.9)** [61] Severe RSV subgroup	—	—
	—	—	—	Adj IR 40.9 (33.4–48.3)*** ^b^ [66] Māori subgroup	—	—
	—	—	—	Adj IR 33.3 (27.2–39.4)*** ^b^ [66] Pacific subgroup	—	—
Economic disadvantage	—	—	—	Adj HR 0.91 (0.63–1.32) [67] Indigenous subgroup Low vs. high disadvantage	—	—
	—	—	—	Adj HR 0.74 (0.63–0.88)* [67] NIHR subgroup Low vs. high disadvantage	—	—
	—	—	—	Adj HR 0.88 (0.82–0.95)* [67] NISR subgroup Low vs. high disadvantage	—	—
	—	—	—	OR 1.2 (0.77–2.0)^a^ [61] Severe RSV subgroup	—	—
Timing of illness onset at hospital presentation	Adj OR 0.78 (0.65–0.94)** [59] Hypoxaemic subgroup	—	—	—	—	—
Trisomy 21^c^	—	—	—	—	—	—
Congenital diaphragmatic hernia^c^	—	—	—	—	—	—
Environmental pollutants^c^	—	—	—	—	—	—

*Note*: Evidence quality: high = green; moderate = yellow; low = orange; very low = red.

Abbreviations: Adj = adjusted for covariates; CI = confidence interval; HR = hazard ratio; IR = incidence rate; IRR = incidence rate ratio; IQR = interquartile range; m = months; NIHR = non‐Indigenous high‐risk; NISR = non‐Indigenous standard‐risk; NR = not reported; OR = odds ratio; PPVS = positive pressure ventilation support; RR = risk ratio; wGA = weeks' gestational age.

^a^ Unadjusted effect estimate (adjusted effect estimate unavailable).

^b^ Seasonal IR per 1000 infants residing in South Auckland, New Zealand, corrected for socioeconomic status.

^c^ Risk factor not reported on in Australasian infants, but reported in international systematic reviews.

* *p* < 0.05.

** *p* < 0.01.

*** *p* < 0.001.

### 3.4 Younger Chronological Age

Three prospective and five retrospective observational studies (*N* = 51 886) investigated younger chronological age as a risk factor [59, 60, 61, 62, 63, 64, 65, 66]. Younger chronological age was associated with an increased likelihood of ICU admission (high to moderate‐quality evidence) [59, 63], mechanical ventilation (high‐quality) [60] and hospital admission for bronchiolitis (high to moderate‐quality) [61, 64, 65, 66]. A secondary analysis of data from a large Australasian RCT (*n* = 1472) found that each increase in 1 week of age decreased the risk of hospital admission by 2% [59].

In a subgroup study of Aboriginal and Torres Strait Islander infants with bronchiolitis, younger chronological age was not found to be an independent risk factor for length of stay (moderate‐quality) [62].

The studies varied in terms of whether age was a continuous or categorical variable, and where categorical, in terms of cut‐off definitions for younger chronological age and comparison groups. These differences, plus the inclusion of studies conducted in subgroups, may explain the variability in effect estimates (Table 3).

### 3.5 Prematurity

Prematurity was investigated as a risk factor in two prospective and four retrospective observational studies (*N* = 18 358) [59, 60, 61, 62, 63, 65]. Prematurity was an independent risk factor for mechanical ventilation (high‐quality) [60], and was associated with increased risk of hospital admission in subgroups of infants with severe RSV infection and NICU graduates (moderate to very low‐quality) [61, 65]. In these studies, a greater degree of prematurity was associated with a higher risk of hospital admission.

There were inconsistent results for the association between prematurity and the risk of ICU admission (moderate‐quality). Prematurity (< 37 weeks' gestational age [wGA]) was associated with an increased risk of ICU admission in one retrospective cohort study, reporting unadjusted effect estimates [63]. However, prematurity was not an independent predictor of ICU admission in a subgroup of infants with bronchiolitis and hypoxaemia [59]. Heterogeneity in the sample populations may explain the inconsistent results.

Prematurity was not significantly associated with hospital length of stay in a subgroup of Aboriginal and Torres Strait Islander infants, based on unadjusted effect estimates from one prospective observational study (low‐quality) [62].

### 3.6 Plural Birth

A large retrospective observational study reported on the association between plural birth and RSV‐associated hospital admission in Indigenous (Aboriginal/Torres Strait Islander) versus non‐Indigenous high‐risk or standard‐risk infants in Australia (*n* = 866 262) [67]. High risk was defined as the presence of bronchopulmonary dysplasia (BPD), low birth weight (< 2500 g), or prematurity (< 37wGA). Plural birth significantly increased the risk of RSV‐associated hospital admission in non‐Indigenous high‐risk (adj HR: 1.20 [95% CI: 1.07–1.35]) and standard‐risk infants (adj HR: 1.47 [95% CI: 1.28–1.70]), including after adjusting for the presence of other risk factors (high‐quality). For Indigenous infants, plural birth was not significantly associated with hospital admission (unadj HR: 1.05 [95% CI: 0.64–1.73], adj HR: not reported).

### 3.7 Faltering Growth/Slow Weight Gain (Failure to Thrive)

Two prospective observational studies investigated faltering growth as a risk factor for severe bronchiolitis (*N* = 1704) [59, 62]. Weight at admission (kg) was not significantly associated with ICU admission in infants with bronchiolitis and hypoxaemia in one secondary analysis of RCT data (moderate‐quality) [59]. In a study of Aboriginal and Torres Strait Islander infants with bronchiolitis, weight at birth (kg) was not significantly associated with hospital length of stay (low‐quality) [62].

### 3.8 Comorbidity

#### 3.8.1 Chronic Lung Disease (CLD)/BPD

Four retrospective observational studies reported on the presence of CLD/BPD as a risk factor (*N* = 16 654) [60, 61, 63, 65]. CLD was associated with a significantly greater likelihood of ICU admission (moderate‐quality) [63]. CLD and BPD were each independent risk factors for mechanical ventilation (moderate‐quality) [60]. In subgroups of NICU graduates and infants with severe RSV, CLD significantly increased the risk of hospital admission (moderate to low‐quality) [61, 65].

#### 3.8.2 Congenital Heart Disease (CHD)

Three retrospective studies investigated CHD as a risk factor (*N* = 13 314) [60, 61, 63]. CHD was associated with a significantly greater risk of ICU admission (low‐quality) [63], mechanical ventilation (in ICU patients; moderate‐quality) [60], and hospital admission (in infants with severe RSV; low‐quality) [61].

#### 3.8.3 Chronic Neurological Condition

Two retrospective studies reported on the presence of a chronic neurological condition as a risk factor (*N* = 13 217) [60, 63]. Infants with bronchiolitis and a chronic neurological condition were found to have a significantly increased risk of ICU admission (low‐quality) [63] and mechanical ventilation (in ICU patients; moderate‐quality) [60].

#### 3.8.4 Any Genetic Disorder

The presence of any genetic disorder was associated with a significantly greater odds of hospital admission in one retrospective study of infants with severe RSV (*n* = 97) (very low‐quality) [61].

#### 3.8.5 Any Comorbidity

The presence of any comorbidity was associated with a greater risk of death and mechanical ventilation in infants with bronchiolitis in one retrospective cohort study (*n* = 604) (moderate‐quality) [68]. The presence of any comorbidity was associated with a longer time on positive pressure ventilation support (+26.8 h), compared to no comorbidity, although it is unclear if the difference was statistically significant (moderate‐quality).

### 3.9 Low Breastfeeding Exposure

There was minimal evidence on the effects of low breastfeeding exposure in Australasian infants with bronchiolitis. We included one prospective observational study in a subgroup of Aboriginal and Torres Strait Islander infants (*n* = 232; median age 5 months (IQR: 3–9)) [62]. This study found no significant difference in the length of stay of Indigenous infants with bronchiolitis who were or were not currently breastfed (low‐quality).

### 3.10 Tobacco Smoke Exposure

Exposure to tobacco smoke was evaluated as a risk factor in one prospective and one retrospective study in Australian infants (*N* = 866 494) [62, 67]. Prenatal exposure to maternal smoking was an independent risk factor for RSV‐associated hospitalisation in Indigenous (adj HR: 1.39 [95% CI: 1.20–1.61]) and non‐Indigenous standard‐risk (adj HR: 1.47 [95% CI: 1.40–1.55]) or high‐risk infants (with prematurity, BPD or low birth weight) (adj HR: 1.26 [95% CI: 1.13–1.41]) (high‐quality) [67]. In a smaller study (*n* = 232) of Aboriginal and Torres Strait Islander infants with bronchiolitis, prenatal exposure to maternal smoking and postnatal exposure to household smoking were not found to affect hospital length of stay (low‐quality) [62]. No studies reported on the effects of vaping exposure.

### 3.11 Indigenous Ethnicity

Two retrospective observational studies in Australian infants reported on Indigenous ethnicity as a risk factor (*N* = 33 133) [61, 64]. Aboriginal or Torres Strait Islander ethnicity was associated with a significantly higher rate of hospital admission for RSV across all age groups (0–2 m, 3–5 m, < 6 m, 6–11 m), compared to non‐Indigenous ethnicity in one large retrospective study (IRR range: 1.6–2.5) (high‐quality) [64]. Aboriginal or Torres Strait Islander ethnicity was an independent predictor of RSV‐associated hospital admission in infants with severe RSV infection (low‐quality) [61].

One prospective observational study found that the seasonal incidence rates for RSV‐associated hospital admission per 1000 infants residing in South Auckland, AoNZ, were significantly higher in Māori (IR: 40.9 [95% CI: 33.4–48.3]) and Pacific infants (IR: 33.3 [95% CI: 27.2–39.4]), compared to infants of Asian (IR: 5.5 [95% CI: 3.6–7.4]) or European/other ethnicity (IR: 9.0 [95% CI: 6.8–11.3]) after controlling for socioeconomic status (moderate‐quality) [66].

### 3.12 Economic Disadvantage

Two retrospective cohort studies evaluated economic disadvantage as a risk factor in Australian infants (*N* = 866 359) [61, 67]. Economic disadvantage was an independent risk factor for RSV‐associated hospital admission in non‐Indigenous infants at otherwise standard or high risk (prematurity, BPD, low birth weight), in one large study (*n* = 866 262) (moderate‐quality) [67]. However, for Aboriginal and Torres Strait Islander infants, the risk of RSV‐associated hospital admission did not vary between those in the most and least disadvantaged quintiles. In one small study (*n* = 97) in infants with severe RSV infection, economic disadvantage was not a significant predictor of hospital admission (low‐quality) [61].

### 3.13 Timing of Illness Onset at Hospital Presentation

One prospective observational study in Australian infants (*N* = 1472) found that those further from their time of illness onset at hospital presentation had significantly lower odds of ICU admission (high‐quality) [59].

### 3.14 Certainty of Evidence Assessment

The certainty of evidence assessments is presented in Appendix S5. The quality of the body of evidence varied between risk factors. The risk factors with high certainty evidence in Australasian infants were younger chronological age, prematurity, plural birth, tobacco smoke exposure, Indigenous ethnicity and timing of illness onset at hospital presentation. The risk factors with moderate certainty evidence were CHD, CLD, chronic neurological conditions, any comorbidity, faltering growth and economic disadvantage. There was low certainty evidence for breastfeeding exposure and very low certainty evidence for any genetic disorder. The evidence quality was downgraded due to concerns about imprecision, inconsistency, indirectness and RoB.

### 3.15 Comparison to International Literature

The risk factors found to be significant risk factors in Australasian infants were consistent with the international literature, aside from breastfeeding exposure and faltering growth [39, 40, 41, 42, 43, 44, 45, 46, 47, 48, 49]. The following risk factors reported in the international literature have not been examined in Australasian infants: trisomy 21 [39], congenital diaphragmatic hernia [44], and exposure to certain environmental pollutants (e.g., sulphur dioxide [SO_2_], nitrogen dioxide [NO_2_], sourced from fossil fuel combustion) [43] (Table 3).

## 4 Discussion

Infants with risk factors for severe bronchiolitis are more likely to deteriorate rapidly and require escalation of care. This systematic review summarised the evidence on risk factors for severe bronchiolitis in Australasian infants. Our findings are largely consistent with the results of systematic reviews using international data to look at the associations of single risk factors with disease severity outcomes in infants with bronchiolitis [39, 40, 41, 42, 43, 44, 45, 46, 47, 48, 49]. Of note, there is international evidence of additional risk factors for severe bronchiolitis beyond those reported in the Australasian data, including the presence of trisomy 21 [39], congenital diaphragmatic hernia [44], and exposure to certain environmental pollutants [43]. These risk factors have yet to be investigated in Australasian populations, although it is likely they would apply.

The quality of the evidence varied between risk factors and by outcomes within risk factors. The best quality evidence was available for younger chronological age, prematurity, tobacco smoke exposure, and Indigenous ethnicity. There was also high‐quality evidence for the timing of illness onset at hospital presentation and plural birth, due to the presence of one large, high‐quality study for each. The lowest quality evidence and, as a result, the risk factor with the greatest degree of uncertainty in the evidence was any genetic disorder.

Although there was low‐quality evidence indicating no significant association between breastfeeding exposure and length of stay in Aboriginal and Torres Strait Islander infants, there is clear international evidence supporting this risk factor [9, 14, 19, 45]. Evidence for this risk factor in our review was only found for a single outcome from a single study. In the international evidence, greater breastfeeding exposure has been shown to be a protective factor against hospital admissions for bronchiolitis. The non‐significant findings for faltering growth are also inconsistent with international evidence, which has shown a significant association between small weight for gestational age (< 10th percentile) and increased odds of hospital admission in infants with bronchiolitis [41]. It is possible that the use of categorical variables with a defined weight cut‐off may have affected the results.

Indigenous ethnicity has been shown to be a risk factor for more severe outcomes from bronchiolitis in Australia and AoNZ [61, 64, 66]. Indigenous ethnicity as a risk factor is attributable to its relationship with other predisposing variables for severe illness, such as the ongoing effects of colonisation and institutional racism in many Australasian hospitals. Addressing these societal and systemic factors would help to improve bronchiolitis outcomes for Māori in AoNZ, and Aboriginal and Torres Strait Islanders in Australia.

Plural or multiple birth was found to be an independent modest risk factor for RSV hospitalisation in non‐Indigenous infants, but not Indigenous infants, in one large, high‐quality Australian study (*n* = 866 262) [67]. International evidence from a systematic review of 12 studies found inconsistent results for this risk factor, with uncertainty regarding the interaction between gestational age and plural birth [46].

**FIGURE 2:**
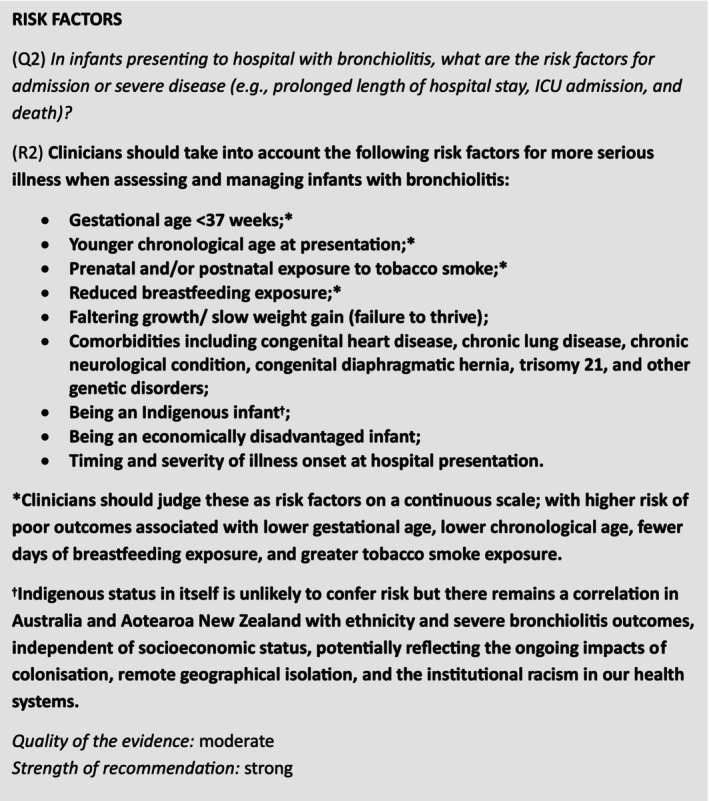
Updated recommendation on risk factors for severe bronchiolitis (Australasian Bronchiolitis Guideline 2025 update).

The findings from this systematic review have informed an update to the Australasian Bronchiolitis Guideline [35], for infants presenting or admitted to hospital with bronchiolitis in Australia or AoNZ (Figure 2). These risk factors should inform the clinical management of infants with bronchiolitis in Australasian hospitals (e.g., decision‐making pertaining to needs for monitoring, admissions, initiation of supportive care or care escalation) and inform targeting of preventative interventions (e.g., monoclonal RSV antibody prophylaxis and hMPV vaccination) in Australasia. Policymakers looking to determine target populations for the delivery of preventative programmes should additionally consider the prevalence of the potentially targeted risk factors from this review, in infants admitted to hospital in Australia and AoNZ.

### 4.1 Limitations

Although there was a large sample size across the included studies (*N* = 895 276), this was contributed to by one especially large study in Australian infants (*N* = 866 262) [67]. There is a relative lack of data from AoNZ compared to Australia, including studies in Māori infants.

Studies were not excluded by the statistical modelling approach. Most (7/10) of the included studies used multivariable models [59, 60, 61, 62, 67, 68], which showed which risk factors were independently associated with disease severity outcomes when taking into account other related variables in the model, or stratified analyses with adjustment for known covariates [66]. In the remaining studies using univariable analyses (3/10), the independent association between the risk factor and severity outcomes is unclear [63, 64, 65].

Our statistical eligibility criteria resulted in the exclusion of four Australasian studies. However, these criteria were used in other bronchiolitis guidelines evaluating risk factors as a method to exclude descriptive studies [69].

Caution needs to be applied in using this review as the sole basis of targeted preventive RSV policies, as data missing from this review includes the overall prevalence of potentially targeted risk factors in infants admitted to hospital in Australia and AoNZ.

### 4.2 Strengths

Bronchiolitis is the most common reason for infants to be admitted to hospital in Australia and AoNZ. For the first time, this systematic review focused on summarising the evidence on risk factors for severe bronchiolitis in Australasian infants specifically, providing clinicians with increased certainty when managing infants.

The included studies reported on data collected across a wide range of years (2001–2016), and although heterogeneous in design, were relatively consistent in identifying risk factors of concern.

### 4.3 Future Research

High‐quality observational studies are needed to investigate risk factors with low‐quality evidence to improve the certainty of evidence. In particular, there is a lack of evidence investigating the association between certain risk factors (e.g., Indigenous ethnicity and economic disadvantage) and critical illness severity outcomes, including ICU admission and mechanical ventilation. The shortage of data in Māori and AoNZ infants should be addressed. Possible new risk factors for severe bronchiolitis, such as vaping exposure, should be investigated.

## 5 Conclusion

This is the first systematic review to identify risk factors for severe bronchiolitis in Australasian infants. The risk factors were largely consistent with the international literature; however, evidence was lacking for some risk factors (e.g., breastfeeding) and possible new risk factors (e.g., vaping exposure). Risk factors should be considered in assessing and managing infants with bronchiolitis in Australasian hospitals and in guiding targeted delivery of immunisation programmes against respiratory viruses (e.g., RSV and hMPV) that may cause bronchiolitis in Australasia.

## Conflicts of Interest

M.L.B., S.R.D., F.B., S.O. and E.O.’s institutions have received equipment from Fisher and Paykel Healthcare to support bronchiolitis research. S.R.D. has received funding from Fisher and Paykel Healthcare for travel to an international meeting discussing high‐flow therapy. The other authors declare no conflicts of interest.

## Supporting information


**Data S1:** Supporting Information.

## Data Availability

Data extraction forms and data can be accessed through request to the corresponding author.
