# Supplementary material for: Risk Factors for Severe Bronchiolitis in Australian and Aotearoa New Zealand Infants: A Systematic Review
Source: J Paediatr Child Health. 2025 Aug 8;61(10):1549–65. doi: 10.1111/jpc.70165 (PMC12515271; doi:10.1111/jpc.70165)
Supplement: Supplementary file 1 — Data S1: Supporting Information. [file JPC-61-1549-s001.docx]

Loveys K, Borland ML, Oakley E, Babl FE, Cotterell E, Haskell L, O’Brien S, Wilson CL, Tavender EJ, Dalziel SR, on behalf of the Paediatric Research in Emergency Departments International Collaborative (PREDICT) Network. Risk factors for severe bronchiolitis in Australian and Aotearoa New Zealand infants: a systematic review. *Journal of Paediatrics and Child Health*. 2025.

# Supplementary material

**Contents**

[Appendix 1. PRISMA 2020 Checklists 2](#_Toc189230467)

[Appendix 2. Systematic search strategies 9](#_Toc189230468)

[Appendix 3. Supplementary methods 30](#_Toc189230469)

[Appendix 4. Study characteristics 31](#_Toc189230470)

[Appendix 5. GRADE certainty of evidence tables 44](#_Toc189230471)

## Appendix 1. PRISMA 2020 Checklists

*From:* Page MJ, McKenzie JE, Bossuyt PM, Boutron I, Hoffmann TC, Mulrow CD, et al. The PRISMA 2020 statement: an updated guideline for reporting systematic reviews. MetaArXiv. 2020, September 14. DOI: 10.31222/osf.io/v7gm2.

**Main Checklist**

| **Topic** | **No.** | **Item** | **Location where item is reported** |
| --- | --- | --- | --- |
| **TITLE** |  |  |  |
| **Title** | 1 | Identify the report as a systematic review. | Title page |
| **ABSTRACT** |  |  |  |
| **Abstract** | 2 | See the PRISMA 2020 for Abstracts checklist |  |
| **INTRODUCTION** |  |  |  |
| **Rationale** | 3 | Describe the rationale for the review in the context of existing knowledge. | Introduction > Paragraph 4 |
| **Objectives** | 4 | Provide an explicit statement of the objective(s) or question(s) the review addresses. | Introduction > Paragraph 4 |
| **METHODS** |  |  |  |
| **Eligibility criteria** | 5 | Specify the inclusion and exclusion criteria for the review and how studies were grouped for the syntheses. | Methods > Study selection > Paragraph 1; Supplementary material > Appendix 3 |
| **Information sources** | 6 | Specify all databases, registers, websites, organisations, reference lists and other sources searched or consulted to identify studies. Specify the date when each source was last searched or consulted. | Methods > Search strategy > Paragraph 1 |
| **Search strategy** | 7 | Present the full search strategies for all databases, registers and websites, including any filters and limits used. | Supplementary Material > Appendix 2 'Systematic search strategies' |
| **Selection process** | 8 | Specify the methods used to decide whether a study met the inclusion criteria of the review, including how many reviewers screened each record and each report retrieved, whether they worked independently, and if applicable, details of automation tools used in the process. | Methods > Study selection > Paragraph 1 |
| **Data collection process** | 9 | Specify the methods used to collect data from reports, including how many reviewers collected data from each report, whether they worked independently, any processes for obtaining or confirming data from study investigators, and if applicable, details of automation tools used in the process. | Methods > Data extraction > Paragraph 1 |
| **Data items** | 10a | List and define all outcomes for which data were sought. Specify whether all results that were compatible with each outcome domain in each study were sought (e.g. for all measures, time points, analyses), and if not, the methods used to decide which results to collect. | Methods > Data extraction > Paragraph 1; Supplementary Material > Appendix 3 |
|  | 10b | List and define all other variables for which data were sought (e.g. participant and intervention characteristics, funding sources). Describe any assumptions made about any missing or unclear information. | Methods > Data extraction > Paragraph 1; Supplementary Material > Appendix 3 |
| **Study risk of bias assessment** | 11 | Specify the methods used to assess risk of bias in the included studies, including details of the tool(s) used, how many reviewers assessed each study and whether they worked independently, and if applicable, details of automation tools used in the process. | Methods > Risk of bias assessment > Paragraph 1 |
| **Effect measures** | 12 | Specify for each outcome the effect measure(s) (e.g. risk ratio, mean difference) used in the synthesis or presentation of results. | Methods > Data synthesis > Paragraph 1 |
| **Synthesis methods** | 13a | Describe the processes used to decide which studies were eligible for each synthesis (e.g. tabulating the study intervention characteristics and comparing against the planned groups for each synthesis (item 5)). | Methods > Data synthesis > Paragraph 1 |
|  | 13b | Describe any methods required to prepare the data for presentation or synthesis, such as handling of missing summary statistics, or data conversions. | Methods > Data synthesis > Paragraph 1 |
|  | 13c | Describe any methods used to tabulate or visually display results of individual studies and syntheses. | Methods > Data synthesis > Paragraph 1 |
|  | 13d | Describe any methods used to synthesize results and provide a rationale for the choice(s). If meta-analysis was performed, describe the model(s), method(s) to identify the presence and extent of statistical heterogeneity, and software package(s) used. | Methods > Data synthesis > Paragraph 1 |
|  | 13e | Describe any methods used to explore possible causes of heterogeneity among study results (e.g. subgroup analysis, meta-regression). | Methods > Data synthesis > Paragraph 1 |
|  | 13f | Describe any sensitivity analyses conducted to assess robustness of the synthesized results. | NA |
| **Reporting bias assessment** | 14 | Describe any methods used to assess risk of bias due to missing results in a synthesis (arising from reporting biases). | Methods > Data synthesis > Paragraph 1 |
| **Certainty assessment** | 15 | Describe any methods used to assess certainty (or confidence) in the body of evidence for an outcome. | Methods > Certainty of evidence assessment > Paragraph 1 |
| **RESULTS** |  |  |  |
| **Study selection** | 16a | Describe the results of the search and selection process, from the number of records identified in the search to the number of studies included in the review, ideally using a flow diagram. | Results > Literature search > Paragraph 1 and Figure 1 |
|  | 16b | Cite studies that might appear to meet the inclusion criteria, but which were excluded, and explain why they were excluded. | Results > Literature search > Paragraph 1 |
| **Study characteristics** | 17 | Cite each included study and present its characteristics. | Results > Literature search > Paragraph 2; Supplementary material > Appendix 4 |
| **Risk of bias in studies** | 18 | Present assessments of risk of bias for each included study. | Results > RoB > Paragraph 1, Table 2 |
| **Results of individual studies** | 19 | For all outcomes, present, for each study: (a) summary statistics for each group (where appropriate) and (b) an effect estimate and its precision (e.g. confidence/credible interval), ideally using structured tables or plots. | Results > Table 3; Supplementary material > Appendix 4 |
| **Results of syntheses** | 20a | For each synthesis, briefly summarise the characteristics and risk of bias among contributing studies. | Results > Risk factors for severe bronchiolitis in Australian infants > see subsection for each risk factor |
|  | 20b | Present results of all statistical syntheses conducted. If meta-analysis was done, present for each the summary estimate and its precision (e.g. confidence/credible interval) and measures of statistical heterogeneity. If comparing groups, describe the direction of the effect. | NA |
|  | 20c | Present results of all investigations of possible causes of heterogeneity among study results. | Results > Risk factors for severe bronchiolitis in Australian infants > see subsection for each risk factor (narrative description as quantitative syntheses were not possible) |
|  | 20d | Present results of all sensitivity analyses conducted to assess the robustness of the synthesized results. | NA |
| **Reporting biases** | 21 | Present assessments of risk of bias due to missing results (arising from reporting biases) for each synthesis assessed. | NA |
| **Certainty of evidence** | 22 | Present assessments of certainty (or confidence) in the body of evidence for each outcome assessed. | Results > Certainty of evidence assessment > Paragraph 1; Supplementary Material > Appendix 5 'GRADE Certainty of evidence tables' |
| **DISCUSSION** |  |  |  |
| **Discussion** | 23a | Provide a general interpretation of the results in the context of other evidence. | Discussion > Paragraphs 1 to 6 |
|  | 23b | Discuss any limitations of the evidence included in the review. | Discussion > Limitations > Paragraphs 1, 2 |
|  | 23c | Discuss any limitations of the review processes used. | Discussion > Limitations > Paragraphs 2, 3 |
|  | 23d | Discuss implications of the results for practice, policy, and future research. | Discussion > Paragraph 6 and Figure 2; Discussion > Strengths > Paragraph 1; Discussion > Future research > Paragraph 1 |
| **OTHER INFORMATION** |  |  |  |
| **Registration and protocol** | 24a | Provide registration information for the review, including register name and registration number, or state that the review was not registered. | Methods > Paragraph 1 |
|  | 24b | Indicate where the review protocol can be accessed, or state that a protocol was not prepared. | Methods > Paragraph 1 |
|  | 24c | Describe and explain any amendments to information provided at registration or in the protocol. | Methods > Paragraph 1 |
| **Support** | 25 | Describe sources of financial or non-financial support for the review, and the role of the funders or sponsors in the review. | Funding statement |
| **Competing interests** | 26 | Declare any competing interests of review authors. | Conflict of interest statement |
| **Availability of data, code and other materials** | 27 | Report which of the following are publicly available and where they can be found: template data collection forms; data extracted from included studies; data used for all analyses; analytic code; any other materials used in the review. | Data availability statement |

**Abstract checklist**

| **Topic** | **No.** | **Item** | **Reported?** |
| --- | --- | --- | --- |
| **TITLE** |  |  |  |
| **Title** | 1 | Identify the report as a systematic review. | Yes |
| **BACKGROUND** |  |  |  |
| **Objectives** | 2 | Provide an explicit statement of the main objective(s) or question(s) the review addresses. | Yes |
| **METHODS** |  |  |  |
| **Eligibility criteria** | 3 | Specify the inclusion and exclusion criteria for the review. | Yes |
| **Information sources** | 4 | Specify the information sources (e.g. databases, registers) used to identify studies and the date when each was last searched. | Yes |
| **Risk of bias** | 5 | Specify the methods used to assess risk of bias in the included studies. | Yes |
| **Synthesis of results** | 6 | Specify the methods used to present and synthesize results. | Yes |
| **RESULTS** |  |  |  |
| **Included studies** | 7 | Give the total number of included studies and participants and summarise relevant characteristics of studies. | Yes |
| **Synthesis of results** | 8 | Present results for main outcomes, preferably indicating the number of included studies and participants for each. If meta-analysis was done, report the summary estimate and confidence/credible interval. If comparing groups, indicate the direction of the effect (i.e. which group is favoured). | Yes |
| **DISCUSSION** |  |  |  |
| **Limitations of evidence** | 9 | Provide a brief summary of the limitations of the evidence included in the review (e.g. study risk of bias, inconsistency and imprecision). | Yes |
| **Interpretation** | 10 | Provide a general interpretation of the results and important implications. | Yes |
| **OTHER** |  |  |  |
| **Funding** | 11 | Specify the primary source of funding for the review. | Yes |
| **Registration** | 12 | Provide the register name and registration number. | Yes |

## Appendix 2. Systematic search strategies

**Ovid MEDLINE(R) ALL <1946 to June 19, 2023>**

Search date: 21/06/23

1 bronchiolitis/ or bronchiolitis, viral/ or respiratory syncytial viruses/ or respiratory syncytial virus, human/ or Respiratory Syncytial Virus Infections/ or (bronchiolit* or wheez* or (Respiratory adj1 Syncytial adj1 Virus*)).af. or rsv.tw. 48765

2 limit 1 to (case reports or comment or editorial or letter) 6402

3 limit 1 to (clinical trial, all or clinical trial, phase i or clinical trial, phase ii or clinical trial, phase iii or clinical trial, phase iv or clinical trial or controlled clinical trial or guideline or meta analysis or practice guideline or randomized controlled trial or "review" or systematic reviews) 8638

4 1 and exp Evidence-Based Medicine/ 135

5 (1 not 2) or 3 or 4 42783

6 *bronchiolitis, viral/bl, ci, cl, co, dg, di, dt, ep, et, ge, hi, im, mi, mo, pa, pp, pc, th, ur, vi 1012

7 *bronchiolitis/bl, ci, cl, co, dg, di, dt, ep, et, ge, hi, im, mi, mo, pa, pp, pc, th, ur, vi 2446

8 6 or 7 3450

9 limit 8 to (case reports or comment or editorial or letter) 805

10 limit 8 to (clinical trial, all or clinical trial, phase i or clinical trial, phase ii or clinical trial, phase iii or clinical trial, phase iv or clinical trial or controlled clinical trial or guideline or meta analysis or practice guideline or randomized controlled trial or "review" or systematic reviews) 877

11 8 and exp Evidence-Based Medicine/ 64

12 (8 not 9) or 10 or 11 2702

13 Natural History/ or exp Epidemiology/ 29277

14 exp "reproducibility of results"/ or (scoring adj1 system*).tw. 503239

15 "severity of illness index"/ or (disease adj1 severity).tw. 311661

16 diagnosis, differential/ 467890

17 physical examination/ or exp auscultation/ or blood pressure determination/ or exp palpation/ or percussion/ or pulse/ or exp vital signs/ 517012

18 exp *Respiratory Tract Infections/ 529915

19 risk factors/ 955289

20 "length of stay"/ or patient admission/ or patient discharge/ or (criteria adj4 discharge).tw. 156283

21 exp intensive care units, pediatric/ or respiratory care units/ or (nicu or icu or picu or intensive-care).tw. 231239

22 morbidity/ or prevalence/ or exp mortality/ 781380

23 exp Diagnostic Imaging/ or radiography, thoracic/ or ((chest adj1 x-ray*) or (chest adj1 xray*)).tw. 2929342

24 exp Hematologic Tests/ or ((blood adj1 test*) or (blood adj1 exam*) or (virologic* adj1 investigation*)).tw. 297077

25 Urinalysis/ 9268

26 Nasal Lavage Fluid/ or ((nose or nasal) adj1 (mucus or mucosa)).tw. 10677

27 Emergency Service, Hospital/ or (emergency adj1 department*).tw. 157373

28 asthma/ or hypersensitivity, immediate/ or (atopy or atopic).tw. 186484

29 (salbutamol or ventolin or levalbuterol or adrenalin* or epinephrin* or (beta* adj2 adrenergic*) or (beta* adj2 agonist*) or ics or (inhaled adj1 corticosteroid*) or montelukast).tw. 133389

30 exp Albuterol/ad, tu 7239

31 exp Epinephrine/ad, tu 10031

32 exp Bronchodilator Agents/ad, tu or bronchodilat*.tw. 63186

33 exp steroids/ad, tu 245236

34 exp Cholinergic Antagonists/ or receptors, adrenergic, beta-2/ or (cholinergic adj1 receptor adj1 block* adj1 agent*).tw. 91309

35 exp Anti-Inflammatory Agents/ 568228

36 exp Adrenal Cortex Hormones/ or (corticosteroid* or (cortico adj1 steroid*) or glucocorticoid* or gluco corticoid*).tw. 513603

37 Leukotriene Antagonists/ or (Leukotriene adj1 receptor adj1 block* adj1 agent*).tw. 3242

38 Saline Solution, Hypertonic/ad, tu or (hypertonic and (saline adj1 solution)).tw. 3206

39 exp Aerosols/ and exp Sodium Chloride/ 410

40 exp "nebulizers and vaporizers"/ and exp sodium chloride/ 116

41 ((aerosoli#ed adj1 saline) or (nebuli#ed adj1 saline)).tw. 203

42 exp Oxygen Inhalation Therapy/ or *Oxygen/ad, st [Administration & Dosage, Standards] 30943

43 (exp Oximetry/ or oximet*.tw.) and (exp "reproducibility of results"/ or (reliability or function or (technical adj1 specification*) or (percutaneous adj1 measurement*)).tw. or exp blood gas analysis/ or ((pulse adj1 oximet*) or (supplementa* adj1 oxygen) or (oxygen adj1 saturation) or (oxygen adj1 therap*) or (oxygen adj1 treatment*)).tw.) 22242

44 continuous positive airway pressure/ or positive pressure respiration/ or (bubble adj1 CPAP).tw. 27198

45 exp Physical Therapy Modalities/ 178123

46 Physical Therapy Specialty/ or physical therapists/ 5873

47 (physiotherap* or (physical adj therap*)).tw. 59298

48 ((nasal* or nose or naso) adj3 suction*).tw. 122

49 suction/ or (deep adj1 suction*).tw. 13138

50 saline.tw. and Administration, Intranasal/ 756

51 ((saline adj1 drop*) or (nasal adj1 saline)).tw. 483

52 exp Fluid Therapy/ or Rehydration Solutions/ 22592

53 enteral nutrition/ or exp parenteral nutrition/ or intubation, gastrointestinal/ 50460

54 (((non adj1 oral) or oral) and (feed* or hydration or fluid* or solution* or therap*)).tw. 211378

55 exp bacterial infections/ 961373

56 exp otitis media/ 25757

57 exp Meningitis/ 58718

58 exp *anti-bacterial agents/tu or (antibiotic* or 3z or aruzilina or atizor or azadose or azasite or azatril or azenil or azibiot or azibiot-neo or azimin or azithral or azithromycin or azitrocin or azitromax or azitromicin or azitromicina or aziwok or azomyne or aztrin or azydrop or azyter or azythromycin or bazyt or cp-62933 or cp-62993 or cp62933 or cp62993 or forcin or inedol or infectoazit or isv-401 or isv401 or kromicin or macrozit or mezatrin or octavax or ordipha or ribotrex or sumamed or sunamed or tobyl or tromix or trozocina or ultreon or vinzam or xithrone or xz-450 or xz450 or zaret or zarom or zetamax or zeto or zibramax or zifin or zimericina or zistic or zithromax or zithrox or zitinn or zitrim or zitrobifan or zitrocin or zitromax or zmax).tw. 490886

59 exp Sepsis/ 141157

60 exp Urinary Tract Infections/ 50669

61 Tracheitis/ 1560

62 (serious adj1 bacterial adj1 infection*).tw. 1294

63 infection control/ or exp primary prevention/ or patient isolation/ 213304

64 COVID-19/ or SARS-CoV-2/ 234537

65 (2019-novel or 2019nCoV or 2019-nCoV or COVID-19 or COVID19 or COVID-2019 or COVID2019 or CONVID-19 or CONVID19 or CORVID-19 or CORVID19 or CoV2 or CoV-2 or HCoV* or Ncov* or Ncorona* or Ncorono* or NcovChina* or NcovChinese* or NcovHubei* or NcovWuhan* or SARS2 or SARS-2 or SARScoronavirus2 or SARScoronavirus-2 or SARScoronovirus2 or SARScoronovirus-2 or SARSCov19 or SARSCov-19 or SARS-CoV-2 or SARSCoV-2 or SARSCoV2 or WN-CoV or WNCoV or wuhan-virus).tw. 341860

66 ((pneumonia* or outbreak* or respiratory-illness* or respiratory-disease* or respiratory-symptom* or seafood-market* or food-market* or wildlife) and (Wuhan or China or Chinese or Hubei or Huanan)).tw. 21170

67 ((new or novel or nouveau or risk factors or "2019" or Wuhan or Hubei or Huanan or China or Chinese) adj3 (coronavirus* or corona virus* or betacoronavirus* or CoV or HCoV)).tw. 75998

68 POST-ACUTE COVID-19 SYNDROME/ or (longCOVID* or postCOVID* or postcoronavirus* or postSARS*).tw. 2221

69 (coronavirus/ or betacoronavirus/ or coronavirus infections/) and (disease outbreaks/ or epidemics/ or pandemics/) 40238

70 ((coronavirus* or corona-virus* or betacoronavirus*) adj3 (pandemic* or epidemic* or outbreak* or crisis)).tw. 15507

71 exp Antibodies, Monoclonal/ or (monoclonal-antibod* or clonal-antibod* or hybridoma-antibod* or nirsevimab or medi-8897 or medi8897 or sp-0232 or sp-232 or sp0232 or sp232 or Motavizumab or medi-524 or medi524 or numax or Palivizumab or abbosynagis or medi-493 or medi493 or synagis or synagys).tw. 362383

72 or/13-71 9536442

73 5 and 72 28221

74 exp pregnant women/ or exp pregnancy/ or prenatal care/ or (parturition or ante-natal or antenatal* or pre-natal* or prenatal* or pregnan*).tw. 1176170

75 exp immunization/ or (immunis* or immuniz* or vaccin*).tw. 554939

76 (Respiratory-syncytial-virus-vaccine* or RSV-vaccine* or Arexvy).tw. 910

77 (74 and 75 and 5) or (74 and 76) 271

78 (75 and 5) or 76 4709

79 12 or 73 or 78 29996

80 (newborn* or new-born* or baby or babies or neonat* or neo-nat* or infan* or toddler* or aged-1 or aged-one or one-year-old or 1-year-old or under-two or under-2 or younger-than-two or younger-than-2 or below-two or below-2 or under-24-months or younger-than-24-months or below-24-months or aged-1-to-23-months or aged-one-to-twenty-three-months).tw,kf,hw. 1692458

81 exp Bronchiolitis Obliterans/ or (bronchiolitis adj1 obliterans).af. 5735

82 (79 and 80) not 81 13211

83 77 not 81 271

84 82 or 83 13251

85 limit 84 to (english language and yr="2000 -Current") 9850

**Embase <1974 to 2023 June 19>**

Search date: 21/06/23

1 bronchiolitis/ or viral bronchiolitis/ or exp human respiratory syncytial virus/ or respiratory syncytial virus infection/ or (bronchiolit* or wheez* or (Respiratory adj1 Syncytial adj1 Virus*)).af. or rsv.tw. 93187

2 limit 1 to (editorial or letter or note) 7059

3 1 and (exp controlled clinical trial/ or exp practice guideline/ or meta analysis/ or "review"/ or "systematic review"/) 16414

4 1 and exp evidence based medicine/ 6467

5 (1 not 2) or 3 or 4 86742

6 *bronchiolitis/co, di, dm, dr, dt, ep, et, pc, rt, th 3796

7 *viral bronchiolitis/co, di, dm, dr, dt, ep, et, pc, th 548

8 6 or 7 4336

9 limit 8 to (editorial or letter or note) 571

10 8 and (exp controlled clinical trial/ or exp practice guideline/ or meta analysis/ or "review"/ or "systematic review"/) 1137

11 8 and exp evidence based medicine/ 555

12 (8 not 9) or 10 or 11 3866

13 History/ or exp Epidemiology/ 4735239

14 reproducibility/ or exp validity/ or scoring system/ or exp reliability/ 847991

15 exp disease severity/ or (severity adj2 illness).tw. 2249656

16 differential diagnosis/ or physical examination/ or palpation/ or percussion/ or blood pressure measurement/ or blood pressure monitoring/ or pulse rate/ or vital sign/ or ascultation.tw. 813575

17 exp *respiratory tract infection/ 235051

18 risk factor/ 1336657

19 "length of stay"/ or hospital admission/ or hospital discharge/ or (criteria adj4 discharge).tw. 638513

20 intensive care unit/ or medical intensive care unit/ or neonatal intensive care unit/ or pediatric intensive care unit/ or (nicu or icu or picu or intensive-care).tw. 452434

21 morbidity/ or prevalence/ or mortality/ or childhood mortality/ or infant mortality/ 2024864

22 exp diagnostic imaging/ or exp thorax radiography/ or ((chest adj1 x-ray*) or (chest adj1 xray*)).tw. 485079

23 exp blood examination/ or ((blood adj1 test*) or (haem* adj1 exam*) or (haem* adj1 test*) or (virologic* adj1 investigation*)).tw. 370894

24 exp urinalysis/ 127990

25 nose mucus/ or nose mucosa/ or (nasal adj1 lavage adj1 fluid*).tw. 18815

26 (emergency adj1 department*).tw. 193543

27 exp asthma/ or atopy/ 315735

28 (salbutamol or ventolin or levalbuterol or adrenalin* or epinephrin* or (beta* adj2 adrenergic*) or (beta* adj2 agonist*) or ics or (inhaled adj1 corticosteroid*) or montelukast).tw. 178202

29 salbutamol/ad, do, dt 18740

30 epinephrine/ad, do, dt 5593

31 exp bronchodilating agent/ad, do, dt or bronchodilat*.tw. 99057

32 exp steroid/ad, do, dt 649505

33 exp cholinergic receptor blocking agent/ or (cholinergic adj1 antagonist*).tw. 318374

34 exp antiinflammatory agent/ 2480733

35 exp corticosteroid/ or (corticosteroid* or (cortico adj1 steroid*) or glucocorticoid* or gluco corticoid*).tw. 1152309

36 exp leukotriene receptor blocking agent/ or (leukotriene adj1 antagonist*).tw. 23668

37 (sodium chloride/ad, do, dt and (aerosol/ or hypertonic solution/ or exp nebulizer/ or vaporizer/)) or (hypertonic and (saline adj1 solution)).tw. 1308

38 ((aerosoli#ed adj1 saline) or (nebuli#ed adj1 saline)).tw. 284

39 exp oximetry/ or exp oxygen therapy/ or oxygen/ad, do 124833

40 (exp oximetry/ or oximet*.tw.) and (reproducibility/ or (reliability or function or (technical adj1 specification*) or (percutaneous adj1 measurement*)).tw. or exp blood gas analysis/ or ((pulse adj1 oximet*) or (supplementa* adj1 oxygen) or (oxygen adj1 saturation) or (oxygen adj1 therap*) or (oxygen adj1 treatment*)).tw.) 24303

41 positive end expiratory pressure ventilation/ or ((continuous adj1 positive adj1 airway adj1 pressure) or (positive adj1 pressure adj1 respiration) or (bubble adj1 CPAP)).tw. 22005

42 exp physiotherapy/ 107415

43 (physiotherap* or physical therap*).tw. 96671

44 ((nasal* or nose or naso) adj3 suction*).tw. 183

45 suction/ or (deep adj1 suction*).tw. 12931

46 sodium chloride/na [Intranasal Drug Administration] 177

47 ((saline adj1 drop*) or (nasal adj1 saline)).tw. 667

48 enteric feeding/ or exp parenteral nutrition/ or exp digestive tract intubation/ 92632

49 (((non adj1 oral) or oral) and (feed* or hydration or fluid* or solution* or therap*)).tw. 337435

50 exp fluid therapy/ or oral rehydration solution/ 112397

51 exp bacterial infection/ 955606

52 exp otitis media/ 38079

53 exp meningitis/ 114158

54 exp *antiinfective agent/dt or (antibiotic* or 3z or aruzilina or atizor or azadose or azasite or azatril or azenil or azibiot or azibiot-neo or azimin or azithral or azithromycin or azitrocin or azitromax or azitromicin or azitromicina or aziwok or azomyne or aztrin or azydrop or azyter or azythromycin or bazyt or cp-62933 or cp-62993 or cp62933 or cp62993 or forcin or inedol or infectoazit or isv-401 or isv401 or kromicin or macrozit or mezatrin or octavax or ordipha or ribotrex or sumamed or sunamed or tobyl or tromix or trozocina or ultreon or vinzam or xithrone or xz-450 or xz450 or zaret or zarom or zetamax or zeto or zibramax or zifin or zimericina or zistic or zithromax or zithrox or zitinn or zitrim or zitrobifan or zitrocin or zitromax or zmax).tw. 977691

55 exp sepsis/ 334492

56 exp urinary tract infection/ 139560

57 exp tracheitis/ 4182

58 (serious adj1 bacterial adj1 infection*).tw. 1891

59 infection control/ or patient care/ or isolation.tw. 787000

60 coronavirus disease 2019/ or experimental coronavirus disease 2019/ 363085

61 (2019-novel or 2019nCoV or 2019-nCoV or COVID-19 or COVID19 or COVID-2019 or COVID2019 or CONVID-19 or CONVID19 or CORVID-19 or CORVID19 or CoV2 or CoV-2 or HCoV* or Ncov* or Ncorona* or Ncorono* or NcovChina* or NcovChinese* or NcovHubei* or NcovWuhan* or SARS2 or SARS-2 or SARScoronavirus2 or SARScoronavirus-2 or SARScoronovirus2 or SARScoronovirus-2 or SARSCov19 or SARSCov-19 or SARS-CoV-2 or SARSCoV-2 or SARSCoV2 or WN-CoV or WNCoV or wuhan-virus).tw. 418425

62 ((pneumonia* or outbreak* or respiratory-illness* or respiratory-disease* or respiratory-symptom* or seafood-market* or food-market* or wildlife) and (Wuhan or China or Chinese or Hubei or Huanan)).tw. 23979

63 ((new or novel or nouveau or ("length of stay" or hospital admission or hospital discharge or (criteria adj4 discharge)) or "2019" or Wuhan or Hubei or Huanan or China or Chinese) adj3 (coronavirus* or corona virus* or betacoronavirus* or CoV or HCoV)).tw. 85970

64 long COVID/ or (longCOVID* or postCOVID* or postcoronavirus* or postSARS*).tw. 4909

65 (coronavirinae/ or betacoronavirus/ or coronavirus infection/) and (epidemic/ or pandemic/) 9531

66 ((coronavirus* or corona-virus* or betacoronavirus*) adj3 (pandemic* or epidemic* or outbreak* or crisis)).tw. 17154

67 severe-acute-respiratory-syndrome-coronavirus-2.hw. 97668

68 coronavirus-disease-2019.hw. 363661

69 exp Monoclonal antibody/ or (monoclonal-antibod* or clonal-antibod* or hybridoma-antibod* or nirsevimab or medi-8897 or medi8897 or sp-0232 or sp-232 or sp0232 or sp232 or Motavizumab or medi-524 or medi524 or numax or Palivizumab or abbosynagis or medi-493 or medi493 or synagis or synagys).tw. 833604

70 or/13-69 14210720

71 5 and 70 69784

72 pregnant woman/ or exp pregnancy/ or prenatal care/ or (parturition or ante-natal or antenatal* or pre-natal* or prenatal* or pregnan*).tw. 1159109

73 exp immunization/ or (immunis* or immuniz* or vaccin*).tw. 656913

74 (Respiratory-syncytial-virus-vaccine* or RSV-vaccine* or Arexvy).tw. 1061

75 (72 and 73 and 5) or (72 and 74) 512

76 (73 and 5) or 74 8119

77 12 or 71 or 76 71881

78 (newborn* or new-born* or baby or babies or neonat* or neo-nat* or infan* or toddler* or aged-1 or aged-one or one-year-old or 1-year-old or under-two or under-2 or younger-than-two or younger-than-2 or below-two or below-2 or under-24-months or younger-than-24-months or below-24-months or aged-1-to-23-months or aged-one-to-twenty-three-months).tw,kf,hw,dq. 1649049

79 bronchiolitis obliterans/ or (bronchiolitis adj1 obliterans).af. 10869

80 (77 and 78) not 79 20025

81 75 not 79 503

82 80 or 81 20142

83 limit 82 to (english language and embase and yr="2000 -Current") 11881

**Cochrane Library**

Search date: 21/06/23

Search Name: Bronchiolitis edited 21.06.23

#1 MeSH descriptor: [Bronchiolitis] explode all trees

#2 bronchiolit* or wheez* or (Respiratory Syncytial Virus*) or rsv

#3 MeSH descriptor: [Respiratory Syncytial Viruses] explode all trees

#4 MeSH descriptor: [Respiratory Syncytial Virus Infections] explode all trees

#5 MeSH descriptor: [Natural History] explode all trees

#6 MeSH descriptor: [Epidemiology] explode all trees

#7 MeSH descriptor: [Severity of Illness Index] explode all trees

#8 MeSH descriptor: [Diagnosis, Differential] explode all trees

#9 MeSH descriptor: [Physical Examination] explode all trees

#10 MeSH descriptor: [Respiratory Tract Infections] explode all trees

#11 MeSH descriptor: [Risk Factors] explode all trees

#12 MeSH descriptor: [Length of Stay] explode all trees

#13 MeSH descriptor: [Patient Admission] explode all trees

#14 MeSH descriptor: [Intensive Care Units] explode all trees

#15 MeSH descriptor: [Morbidity] explode all trees

#16 MeSH descriptor: [Mortality] explode all trees

#17 MeSH descriptor: [Diagnostic Imaging] explode all trees

#18 MeSH descriptor: [Hematologic Tests] explode all trees

#19 MeSH descriptor: [Urinalysis] explode all trees

#20 MeSH descriptor: [Nasal Lavage Fluid] explode all trees

#21 MeSH descriptor: [Emergency Service, Hospital] explode all trees

#22 MeSH descriptor: [Albuterol] explode all trees

#23 MeSH descriptor: [Epinephrine] explode all trees

#24 MeSH descriptor: [Steroids] explode all trees

#25 MeSH descriptor: [Bronchodilator Agents] explode all trees

#26 MeSH descriptor: [Cholinergic Antagonists] explode all trees

#27 MeSH descriptor: [Anti-Inflammatory Agents] explode all trees

#28 MeSH descriptor: [Adrenal Cortex Hormones] explode all trees

#29 MeSH descriptor: [Leukotriene Antagonists] explode all trees

#30 MeSH descriptor: [Saline Solution, Hypertonic] explode all trees

#31 MeSH descriptor: [Aerosols] explode all trees

#32 MeSH descriptor: [Nebulizers and Vaporizers] explode all trees

#33 MeSH descriptor: [Sodium Chloride] explode all trees

#34 (#31 or #32) and #33

#35 MeSH descriptor: [Oxygen Inhalation Therapy] explode all trees

#36 MeSH descriptor: [Oxygen] explode all trees and with qualifier(s): [administration & dosage - AD, standards - ST]

#37 MeSH descriptor: [Oximetry] explode all trees

#38 MeSH descriptor: [Reproducibility of Results] explode all trees

#39 #37 and #38

#40 MeSH descriptor: [Physical Therapy Modalities] explode all trees

#41 MeSH descriptor: [Physical Therapy Specialty] explode all trees

#42 MeSH descriptor: [Suction] explode all trees

#43 MeSH descriptor: [Fluid Therapy] explode all trees

#44 MeSH descriptor: [Infusions, Intravenous] explode all trees

#45 MeSH descriptor: [Administration, Oral] explode all trees

#46 #43 and (#44 or #45)

#47 MeSH descriptor: [Administration, Intranasal] explode all trees

#48 saline (Word variations have been searched)

#49 #48 and #47

#50 MeSH descriptor: [Rehydration Solutions] explode all trees

#51 MeSH descriptor: [Bacterial Infections] explode all trees

#52 MeSH descriptor: [Otitis Media] explode all trees

#53 MeSH descriptor: [Meningitis] explode all trees

#54 MeSH descriptor: [Anti-Bacterial Agents] explode all trees and with qualifier(s): [therapeutic use - TU]

#55 MeSH descriptor: [Sepsis] explode all trees

#56 MeSH descriptor: [Urinary Tract Infections] explode all trees

#57 MeSH descriptor: [Tracheitis] explode all trees

#58 MeSH descriptor: [Radiography, Thoracic] explode all trees

#59 MeSH descriptor: [Asthma] explode all trees

#60 MeSH descriptor: [Hypersensitivity, Immediate] explode all trees

#61 MeSH descriptor: [Receptors, Adrenergic, beta-2] explode all trees

#62 MeSH descriptor: [Continuous Positive Airway Pressure] explode all trees

#63 MeSH descriptor: [Enteral Nutrition] explode all trees

#64 MeSH descriptor: [Parenteral Nutrition] explode all trees

#65 MeSH descriptor: [Intubation, Gastrointestinal] explode all trees

#66 MeSH descriptor: [Infection Control] explode all trees

#67 MeSH descriptor: [Primary Prevention] explode all trees

#68 MeSH descriptor: [Patient Isolation] explode all trees

#69 (History or Epidemiolog* or "severity of illness" or “disease severity” or scoring system* or diagnosis or physical exam* or auscultation or "blood pressure" or palpation* or percussion or pulse or vital sign* or Respiratory Tract Infection* or risk factor* or "length of stay" or admission or discharge or morbidit* or prevalence or mortalit* or "Diagnostic Imaging" or ((chest or thorac*) and (x-ray* or xray* or “x ray” or radiograph*)) or ((Hematolog* or Haematolog* or blood or virolog* or urine) and (Test or tests or exam* or investigation*)) or Urinalys* or “Nasal Lavage” or ((nose or nasal) and (mucosa or mucus)) or emergency department* or asthma* or atopy or atopic or hypersensitiv*) (Word variations have been searched)

#70 salbutamol or albuterol or ventolin or levalbuterol or adrenalin* or epinephrin* or beta2 adrenergic* or beta2 agonist* or ics or corticosteroid* or cortico steroid*OR cortico-steroid* or glucocorticoid* or gluco corticoid* or gluco-corticoid* or montelukast or Bronchodilat* or steroid or steroids or Cholinergic Antagonist* or cholinergic receptor* or Anti Inflammatory Agent* or Adrenal Cortex Hormone* or Leukotriene Antagonist* or Leukotriene receptor* or "Hypertonic Saline" or (("Sodium Chloride" or saline) and (nebuliz* or nebulis* or vaporiz* or vaporis* or aerosol* or intranasal or "intra nasal" or intra-nasal or nasal)) (Word variations have been searched)

#71 (Oxygen or ((Oximetry or oximeter*) and ("reproducibility of results" or reliability or validity or function* or technical specification* or percutaneous measurement* or blood gas analys*)) or CPAP or “continuous positive airway pressure” or “positive pressure respiration” or “positive end respiratory pressure”) (Word variations have been searched)

#72 Physical Therap* or physiotherap* or ((nasal* or nose or naso) and (suction* or toilet or irrigation)) or suction* or saline drop* or "nasal saline" or "intranasal saline" (Word variations have been searched)

#73 (Fluid Therap* or Intravenous infusion* or “non oral” or Rehydrat* or “enteric feeding” or “parenteral nutrition” or “parenteral feeding” or “enteral nutrition” or oral* administ* or bacterial infection* or "otitis media" or Meningitis or antibacterial agent* or anti bacterial agent* or antimicrobial agent* or anti microbial agent* or antibiotic* or 3z or aruzilina or atizor or azadose or azasite or azatril or azenil or azibiot or azibiot-neo or azimin or azithral or azithromycin or azitrocin or azitromax or azitromicin or azitromicina or aziwok or azomyne or aztrin or azydrop or azyter or azythromycin or bazyt or cp-62933 or cp-62993 or cp62933 or cp62993 or forcin or inedol or infectoazit or isv-401 or isv401 or kromicin or macrozit or mezatrin or octavax or ordipha or ribotrex or sumamed or sunamed or tobyl or tromix or trozocina or ultreon or vinzam or xithrone or xz-450 or xz450 or zaret or zarom or zetamax or zeto or zibramax or zifin or zimericina or zistic or zithromax or zithrox or zitinn or zitrim or zitrobifan or zitrocin or zitromax or zmax or Sepsis or septic or Urinary Tract Infection* or tracheitis or serious bacterial infection* or “infection control” or “primary prevention” or isolation or “patient care”) (Word variations have been searched)

#74 “intensive care” or ICU or "respiratory care" or NICU or PICU

#75 MeSH descriptor: [SARS-CoV-2] this term only

#76 MeSH descriptor: [COVID-19] this term only

#77 ((corona* or corono*) NEAR (virus* or viral* or virinae*)) (Word variations have been searched)

#78 (coronavirus* or coronovirus* or coronavirinae* or CoV or CoV2 or CoV-2 or HCoV*) (Word variations have been searched)

#79 (“2019 nCoV” or 2019nCoV or nCoV2019 or nCoV-2019 or COVID-19 or COVID19 or CORVID-19 or CORVID19 or WNCoV or HCoV-19 or HCoV19 or (2019 NEXT novel*) or Ncov or SARSCoV-2 or SARSCoV2 or SARSCov19 or SARSCov-19 or Ncov or Ncorona* or Ncorono* or NcovWuhan* or NcovHubei* or NcovChina* or NcovChinese* or SARS2 or SARS-2 or SARScoronavirus2 or SARScoronavirus-2 or SARScoronovirus2 or SARScoronovirus-2)

#80 (respiratory* NEAR/2 (symptom* or disease* or illness* or condition*) NEAR/10 (Wuhan* or Hubei* or China* or Chinese* or Huanan*))

#81 ((seafood-market* or food-market* or pneumonia*) NEAR/10 (Wuhan* or Hubei* or China* or Chinese* or Huanan*))

#82 ((outbreak* or wildlife* or pandemic* or epidemic*) NEAR (Wuhan* or Hubei or China* or Chinese* or Huanan*))

#83 (COVID-2019 or COVID2019)

#84 MeSH descriptor: [Post-Acute COVID-19 Syndrome] this term only

#85 (longCOVID* or postCOVID* or postcoronavirus* or postSARS*)

#86 MeSH descriptor: [Antibodies, Monoclonal] explode all trees

#87 (monoclonal-antibod* or clonal-antibod* or hybridoma-antibod* or nirsevimab or medi-8897 or medi8897 or sp-0232 or sp-232 or sp0232 or sp232 or Motavizumab or medi-524 or medi524 or numax or Palivizumab or abbosynagis or medi-493 or medi493 or synagis or synagys)

#88 #5 or #6 or #7 or #8 or #9 or #10 or #11 or #12 or #13 or #14 or #15 or #16 or #17 or #18 or #19 or #20 or #21 or #22 or #23 or #24 or #25 or #26 or #27 or #28 or #29 or #30 or #34 or #35 or #36 or #39 or #40 or #41 or #42 or #46 or #49 or #50 or #51 or #52 or #53 or #54 or #55 or #56 or #57 or #58 or #59 or #60 or #61 or #62 or #63 or #64 or #65 or #66 or #67 or #68 or #69 or #70 or #71 or #72 or #73 or #74 or #75 or #76 or #77 or #78 or #79 or #80 or #81 or #82 or #83 or #84 or #85 or #86 or #87

#89 (#1 or #2 or #3 or #4) and #88

#90 MeSH descriptor: [Pregnant Women] explode all trees

#91 MeSH descriptor: [Pregnancy] explode all trees

#92 MeSH descriptor: [Prenatal Care] this term only

#93 (parturition or ante-natal or antenatal* or pre-natal* or prenatal* or pregnan*)

#94 #90 OR #91 or #92 or #93

#95 MeSH descriptor: [Immunization] explode all trees

#96 (immunis* or immuniz* or vaccin*)

#97 #95 OR #96

#98 Respiratory-syncytial-virus-vaccine* or RSV-vaccine* or Arexvy

#99 (#94 and #97 and (#1 or #2 or #3 or #4)) or (#94 and #98)

#100 (#97 and (#1 or #2 or #3 or #4)) or #98

#101 #89 or #100

#102 (newborn* or new born* or baby or babies or neonat* or neo nat* or infan* or toddler* or “aged 1” or “aged one” or “one year old” or “1 year old” or “under two” or “under 2” or “younger than two” or “younger than 2” or “below two” or “below 2” or “under 24 months” or “younger than 24 months” or “below 24 months” or “aged 1 to 23 months” or “aged one to twenty three months”)

#103 MeSH descriptor: [Bronchiolitis Obliterans] explode all trees

#104 ("bronchiolitis obliterans")

#105 (#101 and #102) not (#103 or #104)

#106 #99 not (#103 or #104)

#107 #105 or #106 with Cochrane Library publication date from Jan 2000 to present

**Results = 2164**

**CINAHL**

Search date 21/06/23

S20 S18 AND S19

Limiters - English; Published Date: 20000101-; Peer Reviewed (56)

S19 newborn* or new-born* or baby or babies or neonat* or neo-nat* or infan* or toddler* or aged-1 or aged-one or one-year-old or 1-year-old or under-two or under-2 or younger-than-two or younger-than-2 or below-two or below-2 or under-24-months or younger-than-24-months or below-24-months or aged-1-to-23-months or aged-one-to-twenty-three-months (553,820)

S18 S9 AND S17 (114)

S17 S10 OR S11 OR S12 OR S13 OR S14 OR S15 OR S16 (202,865)

S16 (saline W1 drop*) or (nasal W1 saline) (104)

S15 (MH "Administration, Intranasal") and saline (145)

S14 physiotherap* or "physical therap*" (87,362)

S13 (MH "Physical Therapy+") (159,654)

S12 (MH "Suctioning, Nasopharyngeal") OR (MH "Suction") OR (deep W1 suction*) OR ((nasal* or nose or naso) W3 suction*) (2,755)

S11 (MH "Rehabilitation, Pulmonary")

Limiters - Published Date: 20000101-20011231 (106)

S10 (MH "Chest Physiotherapy (Saba CCC)") OR (MH "Chest Physiotherapy (Iowa NIC)") OR (MH "Chest Physical Therapy+") (870)

S9 (s8 not s5) or s6 or s7 (8,657)

S8 S1 OR S2 OR S3 OR S4 (10,610)

S7 (S1 OR S2 OR S3 OR S4) and (MH "Professional Practice, Evidence-Based+") (108)

S6 S1 OR S2 OR S3 OR S4

Limiters - Publication Type: Clinical Trial, Critical Path, Meta Analysis, Practice Guidelines, Randomized Controlled Trial, Review, Systematic Review (1,501)

S5 S1 OR S2 OR S3 OR S4

Limiters - Publication Type: Case Study, Commentary, editorial, Letter (2,006)

S4 bronchiolit* or wheez* or "respiratory syncytial virus*" or rsv (10,438)

S3 (MH "Respiratory Syncytial Virus Infections") OR (MH "Respiratory Syncytial Viruses") (2,772)

S2 (MH "Bronchial Diseases")

Limiters - Published Date: 20000101-20001231 (40)

S1 (MH "Bronchiolitis+") (2,540)

**PubMed**

Search date 21/6/23

#1 bronchiolitis OR bronchiolitic OR respiratory-syncytial-virus* OR wheez* OR rsv

#2 History OR Epidemiolog* OR "severity of illness" OR "disease severity" OR "scoring system" OR diagnosis OR physical-exam* OR auscultation OR "blood pressure" OR palpation* OR percussion OR pulse OR vital-sign* OR Respiratory-Tract-Infection* OR risk-factor* OR "length of stay" OR admission OR discharge OR "intensive care" OR NICU OR ICU OR PICU OR "respiratory care" OR morbidit* OR prevalence OR mortalit* OR "Diagnostic Imaging" OR ((chest OR thorax OR thoracic) AND (x-ray* OR xray* OR radiograph*)) OR ((Hematolog* OR Haematolog* OR blood OR virolog* OR urine) AND (Test OR tests OR exam* OR investigation*)) OR Urinalys* OR "Nasal Lavage" OR ((nose OR nasal) AND (mucosa OR mucus)) OR emergency-department* OR asthma* OR atopy OR atopic OR hypersensitiv* OR salbutamol OR albuterol OR ventolin OR levalbuterol OR adrenalin* OR epinephrin* OR beta2-adrenergic* OR beta2-agonist* OR ics OR corticosteroid* OR cortico-steroid* OR glucocorticoid* OR gluco corticoid* OR montelukast OR Bronchodilat* OR steroid OR steroids OR Cholinergic-Antagonist* OR cholinergic-receptor* OR Anti-Inflammatory-Agent* OR Adrenal-Cortex-Hormone* OR Leukotriene-Antagonist* OR Leukotriene-receptor* OR "Hypertonic Saline" OR (("Sodium Chloride" OR saline) AND (nebuliz* OR nebulis* OR vaporiz* OR vaporis* OR aerosol* OR intranasal OR intra-nasal OR nasal)) OR Oxygen OR ((Oximetry OR oximeter*) AND ("reproducibility of results" OR reliability OR validity OR function* OR technical-specification* OR percutaneous-measurement* OR blood-gas-analys*)) OR CPAP OR "continuous positive airway pressure" OR "positive pressure respiration" OR "positive end respiratory pressure" OR Physical-Therap* OR physiotherap* OR ((nasal* OR nose OR naso) AND suction*) OR suction* OR saline-drop* OR "nasal saline" OR "nasal toilet" OR "nasal irrigation" OR Fluid-Therap* OR Intravenous-infusion* OR "non oral" OR Rehydrat* OR "enteric feeding" OR "parenteral nutrition" OR "parenteral feeding" OR "enteral nutrition" OR oral* AND administ* OR bacterial-infection* OR "otitis media" OR Meningitis OR antibacterial-agent* OR anti-bacterial-agent* OR antimicrobial-agent* OR anti-microbial-agent* OR antibiotic* OR 3z OR aruzilina OR atizor OR azadose OR azasite OR azatril OR azenil OR azibiot OR azibiot-neo OR azimin OR azithral OR azithromycin OR azitrocin OR azitromax OR azitromicin OR azitromicina OR aziwok OR azomyne OR aztrin OR azydrop OR azyter OR azythromycin OR bazyt OR cp-62933 OR cp-62993 OR cp62933 OR cp62993 OR forcin OR inedol OR infectoazit OR isv-401 OR isv401 OR kromicin OR macrozit OR mezatrin OR octavax OR ordipha OR ribotrex OR sumamed OR sunamed OR tobyl OR tromix OR trozocina OR ultreon OR vinzam OR xithrone OR xz-450 OR xz450 OR zaret OR zarom OR zetamax OR zeto OR zibramax OR zifin OR zimericina OR zistic OR zithromax OR zithrox OR zitinn OR zitrim OR zitrobifan OR zitrocin OR zitromax OR zmax OR Sepsis OR septic OR Urinary-Tract-Infection* OR tracheitis OR serious-bacterial-infection* OR "infection control" OR "primary prevention" OR isolation OR "patient care" OR monoclonal-antibod* OR clonal-antibod* OR hybridoma-antibod* OR nirsevimab OR medi-8897 OR medi8897 OR sp-0232 OR sp-232 OR sp0232 OR sp232 OR Motavizumab OR medi-524 OR medi524 OR numax OR Palivizumab OR abbosynagis OR medi-493 OR medi493 OR synagis OR synagys OR (2019-novel OR 2019nCoV OR 2019-nCoV OR COVID-19 OR COVID19 OR COVID-2019 OR COVID2019 OR CONVID-19 OR CONVID19 OR CORVID-19 OR CORVID19 OR CoV2 OR CoV-2 OR HCoV* OR Ncov* OR Ncorona* OR Ncorono* OR NcovChina* OR NcovChinese* OR NcovHubei* OR NcovWuhan* OR SARS2 OR SARS-2 OR SARScoronavirus2 OR SARScoronavirus-2 OR SARScoronovirus2 OR SARScoronovirus-2 OR SARSCov19 OR SARSCov-19 OR SARS-CoV-2 OR SARSCoV-2 OR SARSCoV2 OR WN-CoV OR WNCoV OR wuhan-virus) OR ((pneumonia* OR outbreak* OR respiratory-illness* OR respiratory-disease* OR respiratory-symptom* OR seafood-market* OR food-market* OR wildlife) AND (Wuhan OR China OR Chinese OR Hubei OR Huanan)) OR ((new OR novel OR nouveau OR 19 OR 2019 OR Wuhan OR Hubei OR Huanan OR China OR Chinese) AND (coronavirus* OR corona virus* OR betacoronavirus* OR CoV OR HCoV)) OR (longCOVID* OR postCOVID* OR postcoronavirus* OR postSARS*) OR ((coronavirus* OR corona-virus* OR betacoronavirus*) AND (pandemic* OR epidemic* OR outbreak* OR crisis))

#3 newborn* OR new-born* OR baby OR babies OR neonat* OR neo-nat* OR infan* OR toddler* OR aged-1 OR aged-one OR one-year-old OR 1-year-old OR under-two OR under-2 OR younger-than-two OR younger-than-2 OR below-two OR below-2 OR under-24-months OR younger-than-24-months OR below-24-months OR aged-1-to-23-months OR aged-one-to-twenty-three-months

#4 NOTNLM

#5 "Bronchiolitis Obliterans"

#6 (#1 AND #2 AND #3 AND #4) NOT #5

Limit 2000 onwards; AND English 2168

[Search name: Cate Wilson 300623 Bronchiolitis AND VOI AND Age AND NLM]

#7 #1 AND #2 AND #3) NOT #5

Limit 2013 onwards; AND English 3576

[Search name: Cate Wilson 300623 Bronchiolitis AND VOI AND Age]

#8 ((bronchiolitis OR bronchiolitic OR respiratory-syncytial-virus* OR wheez* OR rsv) AND (parturition OR ante-natal OR antenatal* OR pre-natal* OR prenatal* OR pregnan*) AND (immunis* OR immuniz* OR vaccin*)) OR ((parturition OR ante-natal OR antenatal* OR pre-natal* OR prenatal* OR pregnan*) AND (Respiratory-syncytial-virus-vaccine* OR RSV-vaccine* OR Arexvy))

#9 (#8 AND #4) NOT #5

Limit 2000 onwards; AND English 160

[Search name: Cate Wilson 300623 Bronchiolitis AND Maternal immunization AND NLM]

#10 #8 NOT #5

Limit 2013 onwards; AND English 271

[Search name: Cate Wilson 300623 Bronchiolitis AND Maternal immunization]

#11 ((bronchiolitis OR bronchiolitic OR respiratory-syncytial-virus* OR wheez* OR rsv) AND (immunis* OR immuniz* OR vaccin*)) OR (Respiratory-syncytial-virus-vaccine* OR RSV-vaccine* OR Arexvy)

#12 (#11 AND #3 AND #4) NOT #5

Limit 2000 onwards; AND English 1087

[Search name: Cate Wilson 300623 Bronchiolitis AND Infant immunization AND Age AND NLM]

#13 (#11 AND #3) NOT #5

Limit 2013 onwards; AND English 1725

[Search name: Cate Wilson 300623 Bronchiolitis AND Infant immunization AND Age]

#14 #6 OR #7 OR #9 OR #10 OR #12 OR #13 4384

## Appendix 3. Supplementary methods

**Search strategy**

The search strategy involved a large systematic search per database that simultaneously covered multiple guideline topics to reduce unneccessary duplication of screening. The searches were structured to include terms on bronchiolitis (e.g., “bronchiolitis/,” “viral bronchiolitis/,” “bronchiolit*”), infants (e.g., “infan*,” “baby”), guideline topics (e.g., “risk factor/”), and outcomes (e.g., “intensive care unit/,” “mortality/,” “infant mortality/,” “hospital admission/”). The results were limited to the English language and by publication date (2000 onwards). The search strategies are presented in Appendix 2.

**Data extraction**

Data were collected on study characteristics (publication year, country, study location, aim, enrolment setting), participant characteristics (sample size, average age, ethnicity, eligibility criteria), risk factor, methodology (study design, outcome measures, analyses), and results. All results related to an outcome were extracted, irrespective of the measure, timepoint, or analysis, provided the result was reported as an adjusted or unadjusted RR, OR, HR, or IRR for dichotomous data, or as a MD or median difference value for continuous data.

**Risk of bias (RoB) assessment**

The Newcastle Ottawa Scale for cohort studies involves assessment of each study for RoB in the selection of cohorts (4 items), comparability of cohorts (1 item), and the outcome assessment (3 items). Each item could be awarded a maximum of one star, apart from the comparability item (up to two stars). Stars are summed to derive a total RoB score per study.

## Appendix 4. Study characteristics

Table 1. Characteristics of the included studies

| **Study design** | **Participants** | **Independent variable** | **Methods** | **Outcomes and results** | **Comments** |
| --- | --- | --- | --- | --- | --- |
| **Butler 2019**  Severe respiratory syncytial virus infection in hospitalized children less than 3 years of age in a temperate and tropical climate. The Pediatric infectious disease journal. 2019 Jan 1;38(1):6-11.  **Country**  Australia  **Study Type**  Retrospective observational  **Location of study**  Women’s and Children’s Hospital, Adelaide, SA; Cairns Hospital Paediatric Department, Carins, QLD, Australia.  **Study Aim**  To determine the factors associated with severe RSV disease. | **Sample**  N=496 children with laboratory-proven RSV infection.  n=97 with severe RSV infection^1^  **Characteristics**  Median age in months (IQR): 5.6 months (IQR 0.6 to 8.0).  Ethnicity:  Aboriginal 12%  Torres Strait Islander 0.4%  Aboriginal and Torres Strait Islander 1.2%  Caucasian 4.8%  Asian 3.4%  African 2.2%  Other/unknown 76%  **Enrolment setting**  ED, hospital ward  **Inclusion criteria**  Age <3 years.  Included children admitted to hospital with a laboratory-confirmed RSV infection between 1 January 2013 and 31 December 2014. | **Risk factor(s)**  Infant age in months.  Prematurity, including <33 weeks gestational age (GA), 33 to 34 +6 weeks GA, and 35 to 37 weeks GA.  Indigenous ethnicity (Aboriginal, Torres Strait Islander).  Socioeconomic disadvantage, defined by SEIFA score (first 5 deciles). SEIFA score is a measure of socioeconomic status based on postcode address.  Comorbidity (reported separately), including:  1) Underlying respiratory disease  2) Underlying cardiac disease  3) Chronic neurologic disease  4) Immunocompromised  5) Chronic metabolic disease  6) Growth disorder  7) Genetic disorder  8) Seizure disorder | Retrospective cohort study with medical chart data.  Univariate logistic regression and multivariate backward stepwise logistic regression were performed.  The multivariate model aimed to identify factors independently predictive of severe RSV hospitalisation. The model included age, gestational age, Indigenous ethnicity, underlying respiratory disease, underlying cardiac disease, and the presence of wheeze on admission. Gestational age <33 weeks and genetic disorders were removed from the model. | **Admission to hospital:** Odds ratio (OR) (95% CI) for hospital admission for severe RSV infection.  1) Age (median 4.7 months (IQR 0.33 to 6.0):  Univariate: OR 0.96 (95% CI 0.92 to 1.0), *p*=.096.  Multivariate: OR 0.95 (95% CI 0.90 to 0.99), ***p*=.02**.  2) Prematurity (<33 weeks GA) (n=15):  Univariate: OR 2.5 (95% CI 1.3 to 5.0), ***p*=.007**.  wGA <33 weeks was not included in the multivariate model.  33 to 34+6 wGA (n=8):  Univariate: OR 1.5 (95% CI 0.66 to 3.6), *p*=.31.  35 to 37 wGA (n=6):  Univariate: OR 0.87 (95% CI 0.35 to 2.2), *p*=.77.  3) Indigenous ethnicity (n=20):  Univariate: OR 1.9 (95% CI 1.1 to 3.4), ***p*=.025**.  Multivariate: OR 2.6 (95% CI 1.4 to 4.9), ***p*=.002**.  4) Socioeconomic disadvantage (n=65):  Univariate: OR 1.2 (95% CI 0.77 to 2.0), *p*=.38.  5) Underlying respiratory disease (n=15):  Univariate: OR 2.6 (95% CI 1.3 to 5.1), ***p*=.006**.  Multivariate: OR 2.6 (95% CI 1.4 to 4.9), ***p*=.029**.  6) Underlying cardiac disease (n=13):  Univariate: OR 3.1 (95% CI 1.5 to 6.4), ***p*=.003**.  Multivariate: OR 2.7 (95% CI 1.1 to 6.4), ***p*=.024**.  7) Other comorbidity (n=9):  Chronic metabolic disease (n=1), immunodeficiency (n=1), growth disorder (n=1), and seizure disorder (n=2) did not significantly predict hospital admission for severe RSV infection in univariate logistic regression analyses (all *p*s >.05).  Presence of a genetic disorder (n=4) was a significant predictor in the univariate analysis, however it was excluded from the multivariate analysis.  OR 4.2 (95% CI 1.0 to 17), ***p*=.045**.  **Primary outcomes:**   - Hospital admission for severe RSV infection;   Plus other secondary outcomes reported in the article. | ^1^ Only this subset of the sample were used in the univariate and multivariate logistic regression models. Severe RSV was determined based on the Brisbane RSV Infection Severity score. |
| **Franklin 2023**  Predictors of Intensive Care Admission in Hypoxemic Bronchiolitis Infants, Secondary Analysis of a Randomized Trial. The Journal of Pediatrics. 2023 May 1;256:92-7.  **Country**  Australia, Aotearoa New Zealand  **Study Type**  Prospective observational (secondary analysis of RCT data)  **Location of study**  17 tertiary and regional hospitals in Australia and Aotearoa New Zealand  **Study Aim**  To evaluate predictors of ICU admission in infants with bronchiolitis, and to assess whether these predictors are equally robust for children receiving high-flow or standard oxygen. | **Sample**  N=1,472 infants with acute bronchiolitis and hypoxaemia  n=152 infants admitted to PICU  **Characteristics**  Median age (IQR): 25.6 weeks (IQR 12.0 to 38.7)  Ethnicity:  Caucasian 52.2%  Pacific Islander 16.6%  Māori 11.7%  Asian 4.1%  Other Indigenous 4%  Arabian 1.6%  African 1.3%  Mixed 2.7%  Other 3.3%  **Enrolment setting**  ED, inpatient units.  **Inclusion criteria**  Age <12 months.  Infants were included who presented to hospital with symptoms of bronchiolitis (per AAP definition), and an oxygen requirement (SpO_2_ <94% in room air at most sites, SpO_2_ <92% at 6 sites).  Infants were excluded who were critically ill with an immediate need for non-invasive or invasive ventilation and ICU admission, or with uncorrected cyanotic heart disease, basal skull fracture, upper airway obstruction, craniofacial malformations, or who received home oxygen. | **Risk factor(s)**  1) Infant age, in weeks.  2) Prematurity, as per PARIS 1 trial definition (<37wGA).  3) Infant weight at admission, in kg.  4) Time of onset of illness to hospital presentation. Log transformed to normalise the distribution, expressed as doubling days. | A pre-planned, secondary analysis of a multicentre RCT (PARIS. 1).  Forward and backwards multivariate logistic regression models were performed to identify independent predictors of ICU admission. | **Admission to ICU:** Adjusted odds ratios (aOR) (95% CI) for intensive care unit admission.  1) Infant age in weeks:  aOR 0.98 (95% CI 0.96 to 0.99), ***p*<.001**.  2) Prematurity (<37wGA):  aOR 1.29 (95% CI 0.77 to 2.14), *p*=.336.  3) Infant weight at admission:  aOR 0.99 (95% CI 0.84 to 1.18), *p*=.217.  4) Time of illness onset at hospital presentation:  aOR 0.78 (95% CI 0.65 to 0.94), ***p*=.009**.  **Primary outcomes:**   - ICU admission;   Plus other secondary outcomes reported in the article. | The allocated intervention (HF therapy vs. standard low-flow oxygen) was not found to modify risk factors for ICU admission. |
| **Homaira 2016**  Risk factors associated with RSV hospitalisation in the first 2 years of life, among different subgroups of children in NSW: a whole-of-population-based cohort study. BMJ open. 2016 Jun 1;6(6):e011398.  **Country**  Australia  **Study Type**  Retrospective cohort  **Location of study**  NSW, Australia  **Study Aim**  To identify risk factors for RSV hospitalisation in different subgroups of high-risk children, compared to the general population. | **Sample**  N=866,262 infants hospitalised with RSV infection.  n=26,523 Indigenous infants  n=66,172 non-Indigenous, high-risk infants  n=773,567 non-Indigenous, standard risk infants  **Characteristics**  Mean age not reported.  Ethnicity:  Indigenous 3%  Non-Indigenous 97%  **Enrolment setting**  NA (population-based dataset)  **Inclusion criteria**  Age ≤24 months.  The study included all children born in NSW from 1 January 2001 to 31 December 2010. | **Risk factor(s)**  1) Socioeconomic disadvantage, according to SEIFA IRSAD score. Category 1 (most disadvantaged) was used as the referent group.  2) Maternal smoking during pregnancy.  3) Plural birth. | Retrospective cohort, whole-of-population-based study, using population-based linked administrative data from the Perinatal Data Collection, Admitted Patient Data Collection, and the Neonatal Intensive Care Units’ Data Collection.  The cohort was divided to compare Indigenous infants (I) to non-Indigenous high-risk (NIHR) and non-Indigenous standard risk infants (NISR).  Indigenous ethnicity was defined as children of mothers whose Indigenous status was recorded as Aboriginal and/or Torres Strait Islander in any of the datasets.  Non-Indigenous, high-risk infants were either born pre-term (<37 wGA), were born at term with a birth weight of <2500g, or had BPD.  Univariate and multivariable analyses were performed. A multivariable model identified independent risk factors for hospital admission. | **Admission to hospital:** Adjusted hazard ratios (Adj HR) (95% CI) for RSV-associated hospitalisation.  I vs. NIHR vs. NISR:  1) Socioeconomic disadvantage (least vs. most disadvantaged):  I: Adj HR 0.91 (95% CI 0.63 to 1.32).  NIHR: Adj HR 0.74 (95% CI 0.63 to 0.88).  NISR: Adj HR 0.88 (95% CI 0.82 to 0.95).  2) Maternal smoking during pregnancy:  I: Adj HR 1.39 (95% CI 1.20 to 1.61).  NIHR: Adj HR 1.26 (95% CI 1.13 to 1.41).  NISR: Adj HR 1.47 (95% CI 1.40 to 1.55).  Statistically significant across all groups (*p*<.05).  3) Plural birth:  I: Unadj HR 1.05 (95% CI 0.64 to 1.73) (Adj HR not reported).  NIHR: Adj HR 1.20 (95% CI 1.07 to 1.35).  NISR: Adj HR 1.47 (95% CI 1.28 to 1.70).  **Primary outcomes:**   - Any episode of RSV-coded hospitalisation in the first two years of life;   Plus other secondary outcomes reported in the article. | Multiparity of the mother, male sex, and being born during the first half of the RSV season were significant predictors of RSV-related hospital admission across all groups (*p*<.05). |
| **McCallum 2016**  Risk factors for adverse outcomes of Indigenous infants hospitalized with bronchiolitis. Pediatric pulmonology. 2016 Jun;51(6):613-23.  **Country**  Australia  **Study Type**  Prospective observational (secondary analysis of three studies)  **Location of studies**  Royal Darwin Hospital, Darwin, NT, Australia.  **Study Aim**  To examine what factors at admission were associated with prolonged length of stay, plus three other aims. | **Sample**  N=232 Indigenous infants with acute bronchiolitis  **Characteristics**  Median age in months (IQR): 5 months (IQR 3 to 9)  Ethnicity:  Aboriginal/ Torres Strait Islander 100%  **Enrolment setting**  Hospital ward.  **Inclusion criteria**  Age ≤24 months.  Indigenous infants hospitalised with bronchiolitis, residing in Darwin.  Infants were excluded with very severe disease (ICU admission), chronic lung disease, congenital heart disease, contraindications to macrolide use, received macrolides in the past 7 days, or with diarrhoea, or clinical or radiological features consistent with pneumonia. | **Risk factor(s)**  1) Infant age, in months.  2) Gestational age, in weeks.  3) Birth weight, in kg.  4) Currently breastfed, yes vs. no.  5) Mother smoked during pregnancy, yes vs. no.  6) Exposure to household smoke, yes vs. no. | Data were combined from three prospective studies: two RCTs and one cohort study.  This study was conducted in a subgroup of Indigenous infants.  Univariate and multivariable analyses were performed. Only significant variables from the univariate analyses were assessed in multivariable analyses. | **Length of stay:** Median difference in length of stay in hours (95% CI), from admission to judged ready for discharge.  1) Infant age:  Univariate: Median difference -1.2 hours (95% CI -2.1 to -0.2), ***p*=.02**.  Multivariable: Median difference -0.6 hours (95% CI -1.6 to 0.5), *p*=.3.  2) Gestational age:  Univariate: Median difference 0.7 hours (95% CI -0.7 to 2.0), *p*=.3.  3) Birth weight:  Univariate: Median difference -0.0 hours (95% CI -5.7 to 6.2), *p*=.9.  For the below categorical variables, ‘no’ was the comparator group:  4) Currently breastfed:  Univariate: Median difference 1.9 hours (95% CI -8.9 to 8.0), *p*=.8.  5) Mother smoked during pregnancy:  Univariate: Median difference 1.7 hours (95% CI -6.6 to 11.4), *p*=.7.  6) Household smoke exposure:  Univariate: Median difference 0.2 hours (95% CI -8.0 to 9.8), *p*=.9.  **Primary outcomes:**   - Prolonged length of stay;   Plus other secondary outcomes reported in the article. | Only modified Tal score at admission was predictive of length of stay in the multivariate analyses. |
| **Oakley 2017**  Intensive care unit admissions and ventilation support in infants with bronchiolitis. Emergency Medicine Australasia. 2017 Aug;29(4):421-8.  **Country**  Australia, Aotearoa New Zealand  **Study Type**  Retrospective observational  **Location of study**  7 Australian and Aotearoa New Zealand hospitals (Royal Children’s Hospital, Melbourne; Sunshine Hospital, Melbourne; Monash Medical Centre, Melbourne; Princess Margaret Hospital, Perth; Royal Children’s Hospital, Brisbane; Kidz First Hospital Middlemore, Auckland; Starship Hospital, Auckland).  **Study Aim**  To assess rates of ICU admission, type of ventilation support, and risk factors for ICU admission in infants with bronchiolitis. | **Sample**  N=3,884 infants with acute bronchiolitis (n=3,589 with available data)  n=204 infants were admitted to ICU  n=3,385 infants were not admitted to ICU  **Characteristics**  Mean age in days (SD): 194.6 days (SD 84.7)  Ethnicity not reported.  **Enrolment setting**  ED  **Inclusion criteria**  Aged 2 to 12 months.  Participants were prospectively identified for inclusion through the CRIB study, between 2009 to 2011.  Infants were included who presented to ED with bronchiolitis, including signs of respiratory distress associated with a viral respiratory tract infection.  Infants were excluded if medical records or ICU data were unavailable. | **Risk factor(s)**  1) Infant age, 2 to <6 months vs. ≥6 months.  2) Prematurity (<37wGA).  3) Chronic lung disease.  4) Neurological disorder.  5) Congenital heart disease. | Retrospective review of electronic medical records and Australia and Aotearoa New Zealand Paediatric Intensive Care (ANZPIC) registry data.  Data were analysed using univariate logistic regression. | **Admission to ICU:** Odds ratio (OR) (95% CI) of ICU admission.  ICU vs. non-ICU  1) Infant age 2 to 6 months:  114 of 204 vs. 1565 of 3385  OR 1.5 (95% CI 1.1 to 2.0), ***p*=.007**.  2) Prematurity:  41 of 199 vs. 492 of 3302  OR 1.5 (95% CI 1.0 to 2.1), ***p*=.03**.  3) Chronic lung disease:  22 of 199 vs. 238 of 3356  OR 1.6 (95% CI 1.0 to 2.6), ***p*=.04**.  4) Neurological disorder:  12 of 198 vs. 95 of 3356  OR 2.2 (95% CI 1.2 to 4.1), ***p*=.01**.  5) Congenital heart disease:  28 of 199 vs. 224 of 3357  OR 2.3 (95% CI 1.5 to 3.5), ***p*<.001**.  **Primary outcomes:**   - ICU admission; - Type of ventilation support;   Plus other secondary outcomes reported in the article. | Previous bronchiolitis was also significantly associated with increased ICU admissions. |
| **Pham 2020**  Ten years of severe respiratory syncytial virus infections in a tertiary paediatric intensive care unit. Journal of Paediatrics and Child Health. 2020 Jan;56(1):61-7.  **Country**  Australia  **Study Type**  Retrospective observational  **Location of study**  Royal Children’s Hospital, Melbourne, VIC, Australia.  **Study Aim**  To describe the epidemiology and treatment of RSV infection in a tertiary PICU. | **Sample**  N=604 infants admitted to PICU with acute RSV infection  n=271 infants with any comorbidity  n=333 infants with no comorbidity  **Characteristics**  Median age in days (IQR): 123 days (59 to 390).  Ethnicity not reported.  **Enrolment setting**  PICU  **Inclusion criteria**  Age criteria not reported.  Infants admitted to PICU with RSV infection confirmed via viral testing.  Infants were excluded who were admitted to PICU for post-operative management, or with hospital-acquired RSV, or with incomplete data. | **Risk factor(s)**  1) Presence of any comorbidity. Comorbidities included congenital anomalies or syndromes, and cardiac, endocrine, gastrointestinal, immunological/ haematological/ oncological, metabolic, neurological, prematurity, renal, respiratory, skeletal, and upper airway-related disorders. | Retrospective analysis of prospectively collected data from the Statistics in Intensive Care database.  Multivariable regression analyses were performed to identify risk factors for mechanical ventilation. | **Death:** Adjusted odds ratio (aOR) (95% CI) of mortality.  1) Presence of any comorbidity:  aOR 2.59 (95% CI 1.42 to 4.70), *p* not reported.  **Mechanical ventilation:** Adjusted OR (95% CI) of mechanical ventilation.  1) Presence of any comorbidity:  aOR 1.97 (95% CI 1.39 to 2.79), ***p*<.001**.  **Time on positive pressure ventilation support (HF therapy, CPAP, BiPAP, MV):** Median difference in duration of mechanical ventilation in hours (IQR).  1) Presence of any comorbidity:  Median difference 26.8 hours longer (95% CI NR).  **Primary outcomes:**   - Clinical presentations; - Comorbidity; - Respiratory support required; - Costs; - Outcomes;   Plus other secondary outcomes reported in the article. | Transfer from an external hospital was also found to be a significant predictor of mechanical ventilation. |
| **Prasad 2020**  Respiratory virus-related emergency department visits and hospitalizations among infants in New Zealand. The Pediatric infectious disease journal. 2020 Aug 1;39(8):e176-82.  **Country**  Aotearoa New Zealand  **Study Type**  Prospective observational  **Location of study**  Kidz First Hospital Middlemore, Auckland, Aotearoa New Zealand.  **Study Aim**  To describe the epidemiology and viral aetiology of ARI events in infants discharged from ED and admitted to hospital. | **Sample**  N=5,412 infants presenting to hospital with ARI, in and outside of the surveillance period.  n=1827 infants admitted to hospital with ARI.  n=782 infants were RSV-positive (n=290 were discharged from ED, n=492 were admitted to hospital with RSV).  **Characteristics**  Mean age not reported.  Ethnicity:  Māori 28.9%  Pacific Islander 48.8%  Asian 9.7%  European/other 12.7%  **Enrolment setting**  ED  **Inclusion criteria**  Aged <12 months.  Infants were included who presented to hospital ED with a suspected ARI during the influenza season between 2014 to 2016, and were either discharged home or admitted to hospital. | **Risk factor(s)**  1) Infant age, categorised by:  <3 months  6 to 11 months  2) Indigenous ethnicity, categorised by:  Māori  Pacific  Asian  European/ other  3) Socioeconomic disadvantage, categorised by 1^st^ to 5^th^ quintile (1=least disadvantaged to 5=most disadvantaged). Calculated from an area-level value of neighbourhood deprivation. | Prospective observational study. Data were collected as part of the Southern Hemisphere Influenza Vaccine Effectiveness and Research (SHIVERS) project. | **Admission to hospital:** Rate ratio (RR) (95% CI) for hospital admissions.  RSV subgroup only:  1) Infant age:  <3 months vs. 6-11 months:  RR 2.54 (95% CI 2.10 to 3.06), *p* not reported.  2) Seasonal hospital admission incidence rate (IR) per 1000 infants residing in the study area (South Auckland) (95% CI). RSV subgroup only:  Indigenous ethnicity (corrected for SES):  Māori: IR 40.9 (95% CI 33.4 to 48.3)  Pacific: IR 33.3 (95% CI 27.2 to 39.4)  Asian: IR 5.5 (95% CI 3.6 to 7.4)  European/ other: IR 9.0 (95% CI 6.8 to 11.3)  *p*<.001 for comparison between Māori or Pacific infants and other ethnicities.  3) Socioeconomic disadvantage (corrected for ethnicity):  1 (least disadvantaged): IR 18.0 (95% CI 9.0 to 27.0)  2: IR 20.3 (95% CI 12.9 to 27.6)  3: IR 20.3 (95% CI 13.0 to 27.6)  4: IR 23.0 (95% CI 17.4 to 28.6)  5 (most disadvantaged): IR 25.7 (95% CI 23.2 to 28.2)  *p*>.05.  **Primary outcomes:**   - Hospital admission for acute respiratory infection (ARI);   Plus other secondary outcomes reported in the article. | Rate ratios were not reported for Indigenous ethnicity or socioeconomic disadvantage, which were assessed in the study (only incidence rates per 1000 children were reported for these variables). |
| **Saravanos 2019**  Respiratory syncytial virus‐associated hospitalisations in Australia, 2006–2015. Medical Journal of Australia. 2019 Jun;210(10):447-53.  **Country**  Australia  **Study Type**  Retrospective cohort  **Location of study**  Australia  **Study Aim**  To estimate the rates of RSV-associated hospitalisation across the age spectrum, and to identify groups at particular risk of serious RSV-associated illness. | **Sample**  N=63,814 hospitalisations of any age with RSV-coded hospitalisation (principal diagnosis)  n=33,036 infants aged 0-11m with RSV-coded hospitalisation (principal diagnosis)  n=10,495 Indigenous infants  n=22,541 non-Indigenous infants  **Characteristics**  Mean age not reported.  Ethnicity:  Aboriginal/ Torres Strait Islander 10.5%  **Enrolment setting**  NA  **Inclusion criteria**  The study included all age groups (however only data on infants aged 0-11m were extracted). | **Risk factor(s)**  1) Indigenous ethnicity, Aboriginal or Torres Strait Islander (compared to non-Indigenous Australians).  2) Infant age, categorised by:  0 to 2 months  3 to 5 months  <6 months  6 to 11 months.  Indigenous and non-Indigenous infants were compared for each age group. | Retrospective cohort study of the Australian Institute of Health and Welfare National Hospital Morbidity Database data for all RSV-related hospitalisations in Australia between 2006 and 2015. | **Admission to hospital:** Incidence rate ratio (IRR) (95% CI) for RSV hospitalisation (principal diagnosis).  1) Indigenous vs. non-Indigenous by age:  0 to 2 months: IRR 1.6 (95% CI 1.5 to 1.7)  3 to 5 months: IRR 2.5 (95% CI 2.3 to 2.6)  <6 months: IRR 1.9 (95% CI 1.8 to 2.0)  6 to 11 months: IRR 2.1 (95% CI 2.0 to 2.3).  **Primary outcomes:**   - RSV hospitalisation;   Plus other secondary outcomes reported in the article. | Hospital length of stay and in-hospital deaths were reported, however the data were not extracted for this outcome as it did not meet our statistical reporting criteria. |
| **Schlapbach 2017**  Burden of disease and change in practice in critically ill infants with bronchiolitis. European Respiratory Journal. 2017 Jun 1;49(6).  **Country**  Australia, Aotearoa New Zealand  **Study Type**  Retrospective observational  **Location of study**  All PICUs plus 19 general ICUs in Australia and Aotearoa New Zealand.  **Study Aim**  To describe the population-based admission rate and severity of bronchiolitis in infants in Australia and Aotearoa New Zealand admitted to intensive care. To identify risk factors for invasive mechanical ventilation. To assess trends in admission rates, management, outcomes, and associated direct healthcare costs between 2002 to 2014. | **Sample**  N=9,628 infants admitted to ICU with acute bronchiolitis.  **Characteristics**  Median age in days (IQR):  2002 to 2009: 91 days (IQR 45 to 201)  2010 to 2014: 139 days (IQR 57 to 281).  Ethnicity not reported.  **Enrolment setting**  PICU  **Inclusion criteria**  Age <24 months.  Infants admitted to a PICU or general ICU in Australia or Aotearoa New Zealand between 1 January 2002 to 31 December 2014 with a diagnosis of bronchiolitis.  Infants were excluded with elective admissions and with pre-existing tracheostomies. | **Risk factor(s)**  1) Infant age (days/30).  2) Prematurity (<37wGA).  3) Chronic neurological condition.  4) Chronic respiratory condition.  5) Bronchopulmonary dysplasia.  6) Congenital heart defect. | Multicentre, retrospective observational study in all patients reported to the Australian and Aotearoa New Zealand Paediatric Intensive Care (ANZPIC) registry.  Multivariate analyses (involving a saturated mix-effects logistic regression model clustering by site and adjusted for all variables) were performed to identify independent predictors of intubation and invasive ventilation. | **Mechanical ventilation:** Adjusted odds ratio (aOR) (95% CI) for requiring intubation and invasive ventilation.  1) Infant age (days/30):  aOR 0.97 (95% CI 0.96 to 0.98), ***p*<.001.**  2) Prematurity (<37wGA):  aOR 1.32 (95% CI 1.15 to 1.52), ***p*<.001.**  3) Chronic neurological condition:  aOR 1.72 (95% CI 1.19 to 2.50), ***p*=.004.**  4) Chronic respiratory condition:  aOR 1.59 (95% CI 1.18 to 2.12), ***p*=.002.**  5) Bronchopulmonary dysplasia:  aOR 1.69 (95% CI 1.32 to 2.15), ***p*<.001.**  6) Congenital heart defect:  aOR 1.88 (95% CI 1.54 to 2.29), ***p*<.001.**  **Primary outcomes:**   - Proportion of infants requiring intubation and invasive ventilation;   Plus other secondary outcomes reported in the article. | Interhospital transport, RSV infection, influenza/ parainfluenza infection, and systolic blood pressure were other significant predictors of need for intubation. |
| **Stevenson 2023**  Health service utilisation for acute respiratory infections in infants graduating from the neonatal intensive care unit: a population-based cohort study. BMC pediatrics. 2023 Jul 1;23(1):335.  **Country**  Australia  **Study Type**  Retrospective cohort  **Location of study**  Princess Margaret Hospital for Children, King Edward Memorial Hospital, Perth, WA, Australia.  **Study Aim**  To describe health service utilisation patterns for acute respiratory infection (ARI) in a cohort of all NICU graduates recorded on an administrative NICU database, in terms of ED presentations and hospitalisations during the first 8 years of life. | **Sample**  N=23,784 NICU graduates  n=3,340 NICU graduates  admitted to hospital with bronchiolitis.  **Characteristics**  Mean age not reported. 2625 (78.6%) of the bronchiolitis sample admitted to hospital were aged ≤11 months.  Ethnicity not reported.  **Enrolment setting**  NICU  **Inclusion criteria**  The included cohort was infants born in WA between 1 January 2002 to 31 December 2013, and admitted to NICU at either of the two participating hospitals (“NICU graduates”). Data were collected over the first 8 years of life.  Infants were excluded who died before discharge. | **Risk factor(s)**  1) Infant age at hospital admission, categorised as:  0 to 5 months  6 to 11 months  2) Gestational age, categorised as:  <28w  28-32w  33-36w  ≥37w  3) Presence of chronic lung disease. | Retrospective cohort study using records from the WA NICU database, the Hospital Morbidity Data Collection, the Emergency Department Data Collection, and the Death Registry. Records between the datasets were probabilistically linked by the Western Australia Department of Health.  Incidence rate ratios were calculated by admission, gestational age, and presence of chronic lung disease from Poisson regression by ALRI diagnosis. Only data for the bronchiolitis subgroup were extracted. | **Admission to hospital:** Incidence rate ratios (IRR) (95% CI) for ARI-related hospital admission.  1) Infant age 6 to 11 months (0 to 5 months as reference group):  IRR 0.64 (95% CI 0.59 to 0.69), ***p*<.001.**  2) Gestational age <28w (≥37w as reference group):  IRR 6.78 (95% CI 6.07 to 7.57), ***p*<.001.**  Gestational age 28-32w (≥37w as reference group):  IRR 3.30 (95% CI 2.98 to 3.64), ***p*<.001.**  Gestational age 33-36w (≥37w as reference group):  IRR 1.56 (95% CI 1.44 to 1.70), ***p*<.001.**  3) Chronic lung disease (no CLD as reference group):  IRR 5.12 (95% CI 4.64 to 5.65), ***p*<.001.**  **Primary outcomes:**   - Incidence of ED presentation in the first 8 years of life; - Incidence of hospitalisation in the first 8 years of life;   Plus other secondary outcomes reported in the article. |  |

## Appendix 5. GRADE certainty of evidence tables

**Question:** In infants presenting to hospital with bronchiolitis, what are the risk factors for admission or severe disease (e.g. prolonged length of hospital stay, intensive care unit (ICU) admission, and death)?

**Settings:** ED, hospital ward, ICU

**Bibliography:** Franklin 2023; Homaira 2016; McCallum 2016; Oakley 2017; Pham 2020; Prasad 2020; Schlapbach 2017; Saravanos 2019; Stevenson 2023.

**Risk factor: younger chronological age**

| Quality assessment | | | | | | | No. of patients | Key findings | Quality | Importance |
| --- | --- | --- | --- | --- | --- | --- | --- | --- | --- | --- |
| No. of studies | Design | Risk of bias | Inconsistency | Indirectness | Imprecision | Other considerations |  |  |  |  |
| **Admission to ICU** (Adj OR for ICU admission) | | | | | | | | | | |
| 1^1^ | Prospective observational (secondary analysis of RCT data) | Not serious | NA | Not serious | Not serious | None | 1,472 | Adj OR 0.98 (95% CI 0.96 to 0.99), *p*<.001. | ⊕⊕⊕⊕  High | Critical |
| **Admission to ICU** (OR for ICU admission) | | | | | | | | | | |
| 1^2^ | Retrospective cohort | Serious^3^ | NA | Not serious | Not serious | None | 3,589 | Infant age 2 to 6 months:  OR 1.5 (95% CI 1.1 to 2.0), *p*=.007. | ⊕⊕⊕⊝  Moderate | Critical |
| **Death** | | | | | | | | | | |
| 0 | NA | NA | NA | NA | NA | NA | NA | NA | NA | Critical |
| **Mechanical ventilation** (Adj OR for intubation and MV) | | | | | | | | | | |
| 1^4^ | Retrospective cohort | Not serious | NA | Not serious | Not serious | None | 9,628 | Infant age (days/30):  Adj OR 0.97 (95% CI 0.96 to 0.98), *p*<.001. | ⊕⊕⊕⊕  High | Critical |
| **Admission to hospital** (RR for hospital admission) | | | | | | | | | | |
| 1^5^ | Prospective observational | Serious^6^ | NA | Not serious | Not serious | None | 492 | Infant age <3 months vs. 6-11 months:  RR 2.54 (95% CI 2.10 to 3.06), *p* NR. | ⊕⊕⊕⊝  Moderate | Important |
| **Admission to hospital** (Adj OR for hospital admissions) | | | | | | | | | | |
| 1^7^ | Retrospective cohort | Not serious | NA | Not serious | Not serious | None | 97 | Infant age (median 4.7 months (IQR 0.33 to 6.0):  Adj OR 0.95 (95% CI 0.90 to 0.99), *p*=.02. | ⊕⊕⊕⊕  High | Important |
| **Admission to hospital** (IRR for hospital admissions, Indigenous vs. non-Indigenous infants) | | | | | | | | | | |
| 1^8^ | Retrospective observational | Not serious | NA | Not serious | Not serious | None | 33,036 | 0 to 2 months: IRR 1.6 (95% CI 1.5 to 1.7)  3 to 5 months: IRR 2.5 (95% CI 2.3 to 2.6)  <6 months: IRR 1.9 (95% CI 1.8 to 2.0)  6 to 11 months: IRR 2.1 (95% CI 2.0 to 2.3).  *p* NR. | ⊕⊕⊕⊕  High | Important |
| 1^9^ | Retrospective cohort | Not serious | NA | Serious^10^ | Not serious | None | 3,340 | Infant age 6 to 11 months (0 to 5 months as reference group):  IRR 0.64 (95% CI 0.59 to 0.69), *p*<.001. | ⊕⊕⊕⊝  Moderate | Important |
| **Length of stay** (median difference in hospital length of stay in hours, from admission to ready for discharge) | | | | | | | | | | |
| 1^11^ | Prospective observational | Not serious | NA | Not serious | Serious^12^ | None | 232 | Multivariate: Median difference -0.6 hours (95% CI -1.6 to 0.5), *p*=.3. | ⊕⊕⊕⊝  Moderate | Important |
| **Time on positive pressure ventilation support (HF therapy, CPAP, MV)** | | | | | | | | | | |
| 0 | NA | NA | NA | NA | NA | NA | NA | NA | NA | Important |
| Adj OR= adjusted odds ratio; CI= confidence interval; CPAP= continuous positive airway pressure; HF= high flow; IRR= incidence rate ratio; MV= mechanical ventilation; NA= not applicable; OR= odds ratio (unadjusted); RR= risk ratio (unadjusted).  ^1^ Franklin 2023. In a population of hypoxaemic infants with bronchiolitis.  ^2^ Oakley 2017.  ^3^ Serious risk of bias. Downgraded due to concerns about confounding.  ^4^ Schlapbach 2017 (PICU patients).  ^5^ Prasad 2020.  ^6^ Serious risk of bias. Downgraded due to concerns about the comparability of the cohorts.  ^7^ Butler 2019.  ^8^ Saravanos 2019.  ^9^ Stevenson 2023. In a population of NICU graduates.  ^10^ Serious indirectness. Downgraded due to indirectness in the population. 78.6% of the bronchiolitis sample who were admitted to hospital were aged ≤11 months.  ^11^ McCallum 2016. In a population of Indigenous infants (Aboriginal, Torres Strait Islander).  ^12^ Serious imprecision. Downgraded due to a reasonably small sample size. | | | | | | | | | | |

**Risk factor: prematurity**

| Quality assessment | | | | | | | No. of patients | Key findings | Quality | Importance |
| --- | --- | --- | --- | --- | --- | --- | --- | --- | --- | --- |
| No. of studies | Design | Risk of bias | Inconsistency | Indirectness | Imprecision | Other considerations |  |  |  |  |
| **Admission to ICU** (Adj OR for ICU admission) | | | | | | | | | | |
| 1^1^ | Prospective observational (secondary analysis of RCT data) | Not serious | NA | Not serious | Serious^2^ | None | 1,472 | Adj OR 1.29 (95% CI 0.77 to 2.14), *p*=.336. | ⊕⊕⊕⊝  Moderate | Critical |
| **Admission to ICU** (OR for ICU admission) | | | | | | | | | | |
| 1^3^ | Retrospective cohort | Serious^4^ | NA | Not serious | Not serious | None | 3,589 | Prematurity:  OR 1.5 (95% CI 1.0 to 2.1), *p*=.03 | ⊕⊕⊕⊝  Moderate | Critical |
| **Death** | | | | | | | | | | |
| 0 | NA | NA | NA | NA | NA | NA | NA | NA | NA | Critical |
| **Mechanical ventilation** (Adj OR for intubation and MV) | | | | | | | | | | |
| 1^5^ | Retrospective cohort | Not serious | NA | Not serious | Not serious | None | 9,628 | Prematurity:  Adj OR 1.32 (95% CI 1.15 to 1.52), *p*<.001. | ⊕⊕⊕⊕  High | Important |
| **Admission to hospital** (OR for hospital admissions) | | | | | | | | | | |
| 1^6^ | Retrospective cohort | Serious^7^ | NA | Not serious | Very serious^8^ | None | 97 | Prematurity (<33 weeks GA) (n=15):  Univariate: OR 2.5 (95% CI 1.3 to 5.0), *p*=.007.  <33 WGA was not included in the multivariate model.  33 to 34+6 wGA (n=8):  Univariate: OR 1.5 (95% CI 0.66 to 3.6), *p*=.31.  35 to 37 wGA (n=6):  Univariate: OR 0.87 (95% CI 0.35 to 2.2), *p*=.77. | ⊕⊝⊝⊝  Very low | Important |
| **Admission to hospital** (IRR for hospital admissions) | | | | | | | | | | |
| 1^9^ | Retrospective cohort | Not serious | NA | Serious^10^ | Not serious | None | 3,340 | <28 wGA (≥37 wGA as reference group):  IRR 6.78 (95% CI 6.07 to 7.57), *p*<.001.  28-32 wGA (≥37 wGA as reference group):  IRR 3.30 (95% CI 2.98 to 3.64), *p*<.001.  33-36 wGA (≥37 wGA as reference group):  IRR 1.56 (95% CI 1.44 to 1.70), *p*<.001. | ⊕⊕⊕⊝  Moderate | Important |
| **Length of stay** (median difference in hospital length of stay in hours) | | | | | | | | | | |
| 1^11^ | Prospective observational | Serious^12^ | NA | Not serious | Serious^13^ | None | 232 | Univariate: Median difference 0.7 hours (95% CI -0.7 to 2.0), *p*=.3. | ⊕⊕⊝⊝  Low | Important |
| **Time on positive pressure ventilation support (HF therapy, CPAP, MV)** | | | | | | | | | | |
| 0 | NA | NA | NA | NA | NA | NA | NA | NA | NA | Important |
| Adj OR= adjusted odds ratio; CI= confidence interval; CPAP= continuous positive airway pressure; HF= high flow; IRR= incidence rate ratio; MV= mechanical ventilation; NA= not applicable; OR= odds ratio (unadjusted); wGA= weeks’ gestational age.  ^1^ Franklin 2023.  ^2^ Serious imprecision. Downgraded due to wide 95% Cis.  ^3^ Oakley 2017.  ^4^ Serious risk of bias. Downgraded due to concerns about confounding.  ^5^ Schlapbach 2017 (PICU patients).  ^6^ Butler 2019.  ^7^ Serious risk of bias. Downgraded due to concerns about confounding that were sufficient to lower confidence in the estimate of effect. Only ORs from the univariate analysis were reported for gestational age in this study.  ^8^ Very serious imprecision. Downgraded due to a very small group size and reasonably wide 95% CIs.  ^9^ Stevenson 2023 (NICU graduates).  ^10^ Serious indirectness. Downgraded due to indirectness in the population. 78.6% of the bronchiolitis sample who were admitted to hospital were aged ≤11 months, and the population were NICU graduates.  ^11^ McCallum 2016.  ^12^ Serious risk of bias. Downgraded due to concerns about confounding, as only the results of the univariate analyses were reported for this variable.  ^13^ Serious imprecision. Downgraded due to a reasonably small sample size. | | | | | | | | | | |

**Risk factor: plural birth**

| Quality assessment | | | | | | | No. of patients | Key findings | Quality | Importance |
| --- | --- | --- | --- | --- | --- | --- | --- | --- | --- | --- |
| No. of studies | Design | Risk of bias | Inconsistency | Indirectness | Imprecision | Other considerations |  |  |  |  |
| **Admission to ICU** | | | | | | | | | | |
| 0 | NA | NA | NA | NA | NA | NA | NA | NA | NA | Critical |
| **Death** | | | | | | | | | | |
| 0 | NA | NA | NA | NA | NA | NA | NA | NA | NA | Critical |
| **Mechanical ventilation** | | | | | | | | | | |
| 0 | NA | NA | NA | NA | NA | NA | NA | NA | NA | Critical |
| **Admission to hospital** (Adj HR or HR for RSV-related hospitalisation, Indigenous vs. non-Indigenous high-risk vs. non-Indigenous standard-risk infants) | | | | | | | | | | |
| 1^1^ | Retrospective cohort | Not serious | NA | Not serious | Not serious | None | 866,262 | I: HR 1.05 (95% CI 0.64 to 1.73)^2^.  NIHR: Adj HR 1.20 (95% CI 1.07 to 1.35).  NISR: Adj HR 1.47 (95% CI 1.28 to 1.70). | ⊕⊕⊕⊕  High | Important |
| **Length of stay** | | | | | | | | | | |
| 0 | NA | NA | NA | NA | NA | NA | NA | NA | NA | Important |
| **Time on positive pressure ventilation support (HF therapy, CPAP, MV)** | | | | | | | | | | |
| 0 | NA | NA | NA | NA | NA | NA | NA | NA | NA | Important |
| Adj HR= adjusted hazard ratio; CI= confidence interval; CPAP= continuous positive airway pressure; HF= high flow; HR= hazard ratio (unadjusted); I= Indigenous; MV= mechanical ventilation; NA= not applicable; NIHR= non-Indigenous high risk; NISR= non-Indigenous standard risk.  ^1^ Homaira 2016.  ^2^ Adjusted HR not reported for this subgroup. | | | | | | | | | | |

**Risk factor: faltering growth/ slow weight gain (failure to thrive)**

| Quality assessment | | | | | | | No. of patients | Key findings | Quality | Importance |
| --- | --- | --- | --- | --- | --- | --- | --- | --- | --- | --- |
| No. of studies | Design | Risk of bias | Inconsistency | Indirectness | Imprecision | Other considerations |  |  |  |  |
| **Admission to ICU** (Adj OR for ICU admission) | | | | | | | | | | |
| 1^1^ | Prospective observational (secondary analysis of RCT data) | Not serious | NA | Not serious | Serious^2^ | None | 1,472 | Infant weight at admission:  Adj OR 0.99 (95% CI 0.84 to 1.18), *p*=.217. | ⊕⊕⊕⊝  Moderate | Critical |
| **Death** | | | | | | | | | | |
| 0 | NA | NA | NA | NA | NA | NA | NA | NA | NA | Critical |
| **Mechanical ventilation** | | | | | | | | | | |
| 0 | NA | NA | NA | NA | NA | NA | NA | NA | NA | Critical |
| **Admission to hospital** | | | | | | | | | | |
| 0 | NA | NA | NA | NA | NA | NA | NA | NA | NA | Important |
| **Length of stay** (median difference in hospital length of stay in hours, from admission to ready for discharge) | | | | | | | | | | |
| 1^3^ | Prospective observational | Serious^4^ | NA | Not serious | Serious^5^ | None | 232 | Birth weight:  Univariate: Median difference -0.0 hours (95% CI -5.7 to 6.2), *p*=.9. | ⊕⊕⊝⊝  Low | Important |
| **Time on positive pressure ventilation support (HF therapy, CPAP, MV)** | | | | | | | | | | |
| 0 | NA | NA | NA | NA | NA | NA | NA | NA | NA | Important |
| Adj OR= adjusted odds ratio; CI= confidence interval; CPAP= continuous positive airway pressure; HF= high flow; MV= mechanical ventilation; NA= not applicable.  ^1^ Franklin 2023.  ^2^ Serious imprecision. Downgraded due to wide 95% CIs.  ^3^ McCallum 2016. In a population of Indigenous infants (Aboriginal, Torres Strait Islander).  ^4^ Serious risk of bias. Downgraded due to concerns about confounding, as only the results of the univariate analyses were reported for this variable.  ^5^ Serious imprecision. Downgraded due to a reasonably small sample size. | | | | | | | | | | |

**Risk factor: chronic lung disease/ bronchopulmonary dysplasia**

| Quality assessment | | | | | | | No. of patients | Key findings | Quality | Importance |
| --- | --- | --- | --- | --- | --- | --- | --- | --- | --- | --- |
| No. of studies | Design | Risk of bias | Inconsistency | Indirectness | Imprecision | Other considerations |  |  |  |  |
| **Admission to ICU** (OR of ICU admission) | | | | | | | | | | |
| 1^1^ | Retrospective cohort | Serious^2^ | NA | Not serious | Not serious | None | 3,589 | CLD:  OR 1.6 (95% CI 1.0 to 2.6), *p*=.04. | ⊕⊕⊕⊝  Moderate | Critical |
| **Death** | | | | | | | | | | |
| 0 | NA | NA | NA | NA | NA | NA | NA | NA | NA | Critical |
| **Mechanical ventilation** (Adj OR for intubation and invasive ventilation) | | | | | | | | | | |
| 1^3^ | Retrospective cohort | Not serious | NA | Not serious | Serious^4^ | None | 9,628 | Chronic respiratory condition:  Adj OR 1.59 (95% CI 1.18 to 2.12), *p*=.002.  BPD:  Adj OR 1.69 (95% CI 1.32 to 2.15), *p*<.001. | ⊕⊕⊕⊝  Moderate | Critical |
| **Admission to hospital** (Adj OR for hospital admissions) | | | | | | | | | | |
| 1^5^ | Retrospective cohort | Not serious | NA | Not serious | Very serious^6^ | None | 97 | Underlying respiratory disease present vs. absent:  Multivariate: Adj OR 2.6 (95% CI 1.4 to 4.9), *p*=.029. | ⊕⊕⊝⊝  Low | Important |
| **Admission to hospital** (IRR for hospital admissions) | | | | | | | | | | |
| 1^7^ | Retrospective cohort | Not serious | NA | Serious^8^ | Not serious | None | 3,340 | CLD vs. no CLD:  IRR 5.12 (95% CI 4.64 to 5.65), *p*<.001. | ⊕⊕⊕⊝  Moderate | Important |
| **Length of stay** | | | | | | | | | | |
| 0 | NA | NA | NA | NA | NA | NA | NA | NA | NA | Important |
| **Time on positive pressure ventilation support (HF therapy, CPAP, MV)** | | | | | | | | | | |
| 0 | NA | NA | NA | NA | NA | NA | NA | NA | NA | Important |
| Adj OR= adjusted odds ratio; BPD= bronchopulmonary dysplasia; CI= confidence interval; CLD= chronic lung disease; CPAP= continuous positive airway pressure; HF= high flow; IRR= incidence rate ratio; MV= mechanical ventilation; NA= not applicable; NICU= neonatal intensive care unit; OR= odds ratio (unadjusted).  ^1^ Oakley 2017.  ^2^ Serious risk of bias. Downgraded due to concerns about confounding.  ^3^ Schlapbach 2017.  ^4^ Serious imprecision. Downgraded due to wide 95% CIs.  ^5^ Butler 2019.  ^6^ Very serious imprecision. Downgraded due to a very small sample size and wide 95% CIs.  ^7^ Stevenson 2023. Population of NICU graduates.  ^8^ Serious indirectness. Downgraded due to indirectness in the population. 78.6% of the bronchiolitis sample who were admitted to hospital were aged ≤11 months, and sample was NICU graduates. | | | | | | | | | | |

**Risk factor: congenital heart disease**

| Quality assessment | | | | | | | No. of patients | Key findings | Quality | Importance |
| --- | --- | --- | --- | --- | --- | --- | --- | --- | --- | --- |
| No. of studies | Design | Risk of bias | Inconsistency | Indirectness | Imprecision | Other considerations |  |  |  |  |
| **Admission to ICU** (OR of ICU admission) | | | | | | | | | | |
| 1^1^ | Retrospective cohort | Serious^2^ | NA | Not serious | Serious^3^ | None | 3,589 | CHD vs. no CHD:  OR 2.3 (95% CI 1.5 to 3.5), *p*<.001. | ⊕⊕⊝⊝  Low | Critical |
| **Death** | | | | | | | | | | |
| 0 | NA | NA | NA | NA | NA | NA | NA | NA | NA | Critical |
| **Mechanical ventilation** (Adj OR for intubation and invasive ventilation) | | | | | | | | | | |
| 1^4^ | Retrospective cohort | Not serious | NA | Not serious | Serious^5^ | None | 9,628 | Congenital heart defect:  Adj OR 1.88 (95% CI 1.54 to 2.29), *p*<.001. | ⊕⊕⊕⊝  Moderate | Critical |
| **Admission to hospital** (Adj OR for hospital admissions) | | | | | | | | | | |
| 1^6^ | Retrospective cohort | Not serious | NA | Not serious | Very serious^7^ | None | 97 | Underlying cardiac disease present vs. absent:  Multivariate: Adj OR 2.7 (95% CI 1.1 to 6.4), *p*=.024. | ⊕⊕⊝⊝  Low | Important |
| **Length of stay** | | | | | | | | | | |
| 0 | NA | NA | NA | NA | NA | NA | NA | NA | NA | Important |
| **Time on positive pressure ventilation support (HF therapy, CPAP, MV)** (MD in duration of MV) | | | | | | | | | | |
| 0 | NA | NA | NA | NA | NA | NA | NA | NA | NA | Important |
| Adj OR= adjusted odds ratio; CHD= congenital heart disease; CI= confidence interval; CPAP= continuous positive airway pressure; HF= high flow; MD= mean difference; MV= mechanical ventilation; NA= not applicable; OR= odds ratio (unadjusted).  ^1^ Oakley 2017.  ^2^ Serious risk of bias. Downgraded due to concerns about confounding.  ^3^ Serious imprecision. Downgraded due to wide 95% CIs.  ^4^ Schlapbach 2017.  ^5^ Serious imprecision. Downgraded due to wide 95% CIs.  ^6^ Butler 2019.  ^7^ Very serious imprecision. Downgraded due to a very small sample size and wide 95% CIs. | | | | | | | | | | |

**Risk factor: chronic neurological condition**

| Quality assessment | | | | | | | No. of patients | Key findings | Quality | Importance |
| --- | --- | --- | --- | --- | --- | --- | --- | --- | --- | --- |
| No. of studies | Design | Risk of bias | Inconsistency | Indirectness | Imprecision | Other considerations |  |  |  |  |
| **Admission to ICU** (OR of ICU admission) | | | | | | | | | | |
| 1^1^ | Retrospective cohort | Serious^2^ | NA | Not serious | Serious^3^ | None | 3,589 | OR 2.2 (95% CI 1.2 to 4.1), *p*=.01. | ⊕⊕⊝⊝  Low | Critical |
| **Death** | | | | | | | | | | |
| 0 | NA | NA | NA | NA | NA | NA | NA | NA | NA | Critical |
| **Mechanical ventilation** (Adj OR of intubation and invasive ventilation) | | | | | | | | | | |
| 1^4^ | Retrospective cohort | Not serious | NA | Not serious | Serious^5^ | None | 9,628 | Adj OR 1.72 (95% CI 1.19 to 2.50), *p*=.004. | ⊕⊕⊕⊝  Moderate | Critical |
| **Admission to hospital** | | | | | | | | | | |
| 0 | NA | NA | NA | NA | NA | NA | NA | NA | NA | Important |
| **Length of stay** | | | | | | | | | | |
| 0 | NA | NA | NA | NA | NA | NA | NA | NA | NA | Important |
| **Time on positive pressure ventilation support (HF therapy, CPAP, MV)** | | | | | | | | | | |
| 0 | NA | NA | NA | NA | NA | NA | NA | NA | NA | Important |
| Adj OR= adjusted odds ratio; CI= confidence interval; CPAP= continuous positive airway pressure; HF= high flow; MV= mechanical ventilation; NA= not applicable; OR= odds ratio (unadjusted).  ^1^ Oakley 2017.  ^2^ Serious risk of bias. Downgraded due to concerns about confounding.  ^3^ Serious imprecision. Downgraded due to wide 95% CIs.  ^4^ Schlapbach 2017.  ^5^ Serious imprecision. Downgraded due to wide 95% CIs. | | | | | | | | | | |

**Risk factor: any genetic disorder**

| Quality assessment | | | | | | | No. of patients | Key findings | Quality | Importance |
| --- | --- | --- | --- | --- | --- | --- | --- | --- | --- | --- |
| No. of studies | Design | Risk of bias | Inconsistency | Indirectness | Imprecision | Other considerations |  |  |  |  |
| **Admission to ICU** | | | | | | | | | | |
| 0 | NA | NA | NA | NA | NA | NA | NA | NA | NA | Critical |
| **Death** | | | | | | | | | | |
| 0 | NA | NA | NA | NA | NA | NA | NA | NA | NA | Critical |
| **Mechanical ventilation** | | | | | | | | | | |
| 0 | NA | NA | NA | NA | NA | NA | NA | NA | NA | Critical |
| **Admission to hospital** (number of hospital admissions due to RSV infection) | | | | | | | | | | |
| 1^1^ | Retrospective cohort | Serious^2^ | NA | Not serious | Very serious^3^ | None | 97 | Univariate: OR 4.2 (95% CI 1.0 to 17), *p=.045.* | ⊕⊝⊝⊝  Very low | Important |
| **Length of stay** | | | | | | | | | | |
| 0 | NA | NA | NA | NA | NA | NA | NA | NA | NA | Important |
| **Time on positive pressure ventilation support (HF therapy, CPAP, MV)** | | | | | | | | | | |
| 0 | NA | NA | NA | NA | NA | NA | NA | NA | NA | Important |
| CI= confidence interval; CPAP= continuous positive airway pressure; HF= high flow; MV= mechanical ventilation; NA= not applicable; OR= odds ratio (unadjusted).  ^1^ Butler 2019.  ^2^ Serious risk of bias. Downgraded due to concerns about confounding that were sufficient to lower confidence in the estimate of effect. Only ORs from the univariate analysis were reported for this variable.  ^3^ Very serious imprecision. Downgraded due to a very small sample size and wide 95% CIs. | | | | | | | | | | |

**Risk factor: any comorbidity**

| Quality assessment | | | | | | | No. of patients | Key findings | Quality | Importance |
| --- | --- | --- | --- | --- | --- | --- | --- | --- | --- | --- |
| No. of studies | Design | Risk of bias | Inconsistency | Indirectness | Imprecision | Other considerations |  |  |  |  |
| **Admission to ICU** | | | | | | | | | | |
| 0 | NA | NA | NA | NA | NA | NA | NA | NA | NA | Critical |
| **Death** (Adj OR for mortality) | | | | | | | | | | |
| 1^1^ | Retrospective cohort | Not serious | NA | Not serious | Serious^2^ | None | 604 | Adj OR 2.59 (95% CI 1.42 to 4.70), *p* NR. | ⊕⊕⊕⊝  Moderate | Critical |
| **Mechanical ventilation** (Adj OR for MV) | | | | | | | | | | |
| 1^1^ | Retrospective cohort | Not serious | NA | Not serious | Serious^2^ | None | 604 | Adj OR 1.97 (95% CI 1.39 to 2.79), *p*<.001. | ⊕⊕⊕⊝  Moderate | Critical |
| **Admission to hospital** | | | | | | | | | | |
| 0 | NA | NA | NA | NA | NA | NA | NA | NA | NA | Important |
| **Length of stay** | | | | | | | | | | |
| 0 | NA | NA | NA | NA | NA | NA | NA | NA | NA | Important |
| **Time on positive pressure ventilation support (HF therapy, CPAP, MV)** (median difference in duration of MV in hours) | | | | | | | | | | |
| 1^1^ | Retrospective cohort | Not serious | NA | Not serious | Serious^3^ | None | 604 | Median difference 26.8 hours longer (95% CI NR). | ⊕⊕⊕⊝  Moderate | Important |
| Adj OR= adjusted odds ratio; CI= confidence interval; CPAP= continuous positive airway pressure; HF= high flow; MV= mechanical ventilation; NA= not applicable; NR= not reported.  ^1^ Pham 2020.  ^2^ Serious imprecision. Downgraded due to wide 95% CIs.  ^3^ Serious imprecision. Downgraded as 95% CIs not reported for the comparison. IQRs reported for the estimates from each group were reasonably wide. | | | | | | | | | | |

**Risk factor: low breastfeeding exposure**

| Quality assessment | | | | | | | No. of patients | Key findings | Quality | Importance |
| --- | --- | --- | --- | --- | --- | --- | --- | --- | --- | --- |
| No. of studies | Design | Risk of bias | Inconsistency | Indirectness | Imprecision | Other considerations |  |  |  |  |
| **Admission to ICU** | | | | | | | | | | |
| 0 | NA | NA | NA | NA | NA | NA | NA | NA | NA | Critical |
| **Death** | | | | | | | | | | |
| 0 | NA | NA | NA | NA | NA | NA | NA | NA | NA | Critical |
| **Mechanical ventilation** | | | | | | | | | | |
| 0 | NA | NA | NA | NA | NA | NA | NA | NA | NA | Critical |
| **Admission to hospital** | | | | | | | | | | |
| 0 | NA | NA | NA | NA | NA | NA | NA | NA | NA | Important |
| **Length of stay** (median difference in hospital length of stay in hours, from admission to ready for discharge) | | | | | | | | | | |
| 1^1^ | Prospective observational | Serious^2^ | NA | Not serious | Serious^3^ | None | 232 | Currently breastfed:  Univariate: Median difference 1.9 hours (95% CI -8.9 to 8.0), *p*=.8. | ⊕⊕⊝⊝  Low | Important |
| **Time on positive pressure ventilation support (HF therapy, CPAP, MV)** | | | | | | | | | | |
| 0 | NA | NA | NA | NA | NA | NA | NA | NA | NA | Important |
| ALRI= acute lower respiratory infection; CI= confidence interval; CPAP= continuous positive airway pressure; HF= high flow; MV= mechanical ventilation; NA= not applicable.  ^1^ McCallum 2016. In a population of Indigenous infants (Aboriginal, Torres Strait Islander).  ^2^ Serious risk of bias. Downgraded due to concerns about confounding, as only the results of the univariate analyses were reported for this variable.  ^3^ Serious imprecision. Downgraded due to a reasonably small sample size and wide 95% CIs that include reasonable benefit and harm. | | | | | | | | | | |

**Risk factor: tobacco smoke exposure**

| Quality assessment | | | | | | | No. of patients | Key findings | Quality | Importance |
| --- | --- | --- | --- | --- | --- | --- | --- | --- | --- | --- |
| No. of studies | Design | Risk of bias | Inconsistency | Indirectness | Imprecision | Other considerations |  |  |  |  |
| **Admission to ICU** | | | | | | | | | | |
| 0 | NA | NA | NA | NA | NA | NA | NA | NA | NA | Critical |
| **Death** | | | | | | | | | | |
| 0 | NA | NA | NA | NA | NA | NA | NA | NA | NA | Critical |
| **Mechanical ventilation** | | | | | | | | | | |
| 0 | NA | NA | NA | NA | NA | NA | NA | NA | NA | Critical |
| **Admission to hospital** (Adj HR for hospital admissions, Indigenous vs. non-Indigenous high risk vs. non-Indigenous standard risk) | | | | | | | | | | |
| 1^1^ | Retrospective cohort | Not serious | NA | Not serious | Not serious | None | 866,262 | Maternal smoking during pregnancy vs. no exposure:  I: Adj HR 1.39 (95% CI 1.20 to 1.61).  NIHR: Adj HR 1.26 (95% CI 1.13 to 1.41).  NISR: Adj HR 1.47 (95% CI 1.40 to 1.55).  Statistically significant across all groups (*p*<.05). | ⊕⊕⊕⊕  High | Important |
| **Length of stay** (median difference in hospital length of stay in hours, from admission to ready for discharge) | | | | | | | | | | |
| 1^2^ | Prospective observational | Serious^3^ | NA | Not serious | Serious^4^ | None | 232 | Mother smoked during pregnancy vs. no exposure:  Univariate: Median difference 1.7 hours (95% CI -6.6 to 11.4), *p*=.7. | ⊕⊕⊝⊝  Low | Important |
| 1^2^ | Prospective observational | Serious^3^ | NA | Not serious | Serious^4^ | None | 232 | Household smoke exposure vs. no exposure:  Univariate: Median difference 0.2 hours (95% CI -8.0 to 9.8), *p*=.9. | ⊕⊕⊝⊝  Low | Important |
| **Time on positive pressure ventilation support (HF therapy, CPAP, MV)** | | | | | | | | | | |
| 0 | NA | NA | NA | NA | NA | NA | NA | NA | NA | Important |
| Adj HR= adjusted hazard ratios; CI= confidence interval; CPAP= continuous positive airway pressure; HF= high flow; I= Indigenous; MV= mechanical ventilation; NA= not applicable; NIHR= non-Indigenous high risk; NISR= non-Indigenous standard risk.  ^1^ Homaira 2016.  ^2^ McCallum 2016. In a population of Indigenous infants (Aboriginal, Torres Strait Islander).  ^3^ Serious risk of bias. Downgraded due to concerns about confounding, as only the results of the univariate analyses were reported for this variable.  ^4^ Serious imprecision. Downgraded due to a reasonably small sample size and wide 95% CIs that include reasonable benefit and harm. | | | | | | | | | | |

**Risk factor: Indigenous ethnicity**

| Quality assessment | | | | | | | No. of patients | Key findings | Quality | Importance |
| --- | --- | --- | --- | --- | --- | --- | --- | --- | --- | --- |
| No. of studies | Design | Risk of bias | Inconsistency | Indirectness | Imprecision | Other considerations |  |  |  |  |
| **Admission to ICU** | | | | | | | | | | |
| 0 | NA | NA | NA | NA | NA | NA | NA | NA | NA | Critical |
| **Death** | | | | | | | | | | |
| 0 | NA | NA | NA | NA | NA | NA | NA | NA | NA | Critical |
| **Mechanical ventilation** | | | | | | | | | | |
| 0 | NA | NA | NA | NA | NA | NA | NA | NA | NA | Critical |
| **Admission to hospital** (OR for hospital admissions) | | | | | | | | | | |
| 1^1^ | Retrospective cohort | Not serious | NA | Not serious | Very serious^2^ | None | 97 | Multivariate: OR 2.6 (95% CI 1.4 to 4.9), *p*=.002. | ⊕⊕⊝⊝  Low | Important |
| **Admission to hospital** (IRR for RSV hospitalisation, Indigenous vs. non-Indigenous infants) | | | | | | | | | | |
| 1^3^ | Retrospective observational | Not serious | NA | Not serious | Not serious | None | 33,036 | 0 to 2 months: IRR 1.6 (95% CI 1.5 to 1.7)  3 to 5 months: IRR 2.5 (95% CI 2.3 to 2.6)  <6 months: IRR 1.9 (95% CI 1.8 to 2.0)  6 to 11 months: IRR 2.1 (95% CI 2.0 to 2.3). | ⊕⊕⊕⊕  High | Important |
| **Admission to hospital** (seasonal IR per 1000 infants residing in South Auckland, corrected for SES) | | | | | | | | | | |
| 1^4^ | Prospective observational | Serious^5^ | NA | Not serious | Not serious | None | 492 | Māori: IR 40.9 (95% CI 33.4 to 48.3)  Pacific: IR 33.3 (95% CI 27.2 to 39.4)  Asian: IR 5.5 (95% CI 3.6 to 7.4)  European/ other: IR 9.0 (95% CI 6.8 to 11.3)  *p*<.001 for comparison between Māori and Pacific infants to Asian and European/ other infants. | ⊕⊕⊕⊝  Moderate | Important |
| **Length of stay** | | | | | | | | | | |
| 0 | NA | NA | NA | NA | NA | NA | NA | NA | NA | Important |
| **Time on positive pressure ventilation support (HF therapy, CPAP, MV)** | | | | | | | | | | |
| 0 | NA | NA | NA | NA | NA | NA | NA | NA | NA | Important |
| CI= confidence interval; CPAP= continuous positive airway pressure; HF= high flow; IR=incidence rate; IRR= incidence rate ratio; MV= mechanical ventilation; NA= not applicable; OR= odds ratio; SES= socioeconomic status.  ^1^ Butler 2019.  ^2^ Very serious imprecision. Downgraded due to a very small sample size and wide 95% CIs.  ^3^ Saravanos 2019.  ^4^ Prasad 2020.  ^5^ Serious risk of bias. Downgraded due to concerns about the comparability of the cohorts. | | | | | | | | | | |

**Risk factor: economic disadvantage**

| Quality assessment | | | | | | | No. of patients | Key findings | Quality | Importance |
| --- | --- | --- | --- | --- | --- | --- | --- | --- | --- | --- |
| No. of studies | Design | Risk of bias | Inconsistency | Indirectness | Imprecision | Other considerations |  |  |  |  |
| **Admission to ICU** | | | | | | | | | | |
| 0 | NA | NA | NA | NA | NA | NA | NA | NA | NA | Critical |
| **Death** | | | | | | | | | | |
| 0 | NA | NA | NA | NA | NA | NA | NA | NA | NA | Critical |
| **Mechanical ventilation** | | | | | | | | | | |
| 0 | NA | NA | NA | NA | NA | NA | NA | NA | NA | Critical |
| **Admission to hospital** (OR for hospital admissions) | | | | | | | | | | |
| 1^1^ | Retrospective cohort | Serious^2^ | NA | Not serious | Serious^3^ | None | 97 | Univariate: OR 1.2 (95% CI 0.77 to 2.0), *p*=.38. | ⊕⊕⊝⊝  Low | Important |
| **Admission to hospital** (Adj HR for hospital admissions, in least vs. most disadvantaged quintiles) | | | | | | | | | | |
| 1^4^ | Retrospective cohort | Not serious | NA | Not serious | Serious^5^ | None | 866,262 | I: Adj HR 0.91 (95% CI 0.63 to 1.32).  NIHR: Adj HR 0.74 (95% CI 0.63 to 0.88).  NISR: Adj HR 0.88 (95% CI 0.82 to 0.95).  Statistically significant in NIHR and NISR groups (*p*<.05). | ⊕⊕⊕⊝  Moderate | Important |
| **Admission to hospital** (seasonal IR for hospital admissions per 1000 infants residing in South Auckland, adjusted for ethnicity) | | | | | | | | | | |
| 1^6^ | Prospective cohort | Serious^7^ | NA | Not serious | Not serious | None | 492 | Socioeconomic disadvantage (corrected for ethnicity):  1 (least disadvantaged): IR 18.0 (95% CI 9.0 to 27.0)  2: IR 20.3 (95% CI 12.9 to 27.6)  3: IR 20.3 (95% CI 13.0 to 27.6)  4: IR 23.0 (95% CI 17.4 to 28.6)  5 (most disadvantaged): IR 25.7 (95% CI 23.2 to 28.2)  *p*>.05. | ⊕⊕⊕⊝  Moderate | Important |
| **Length of stay** | | | | | | | | | | |
| 0 | NA | NA | NA | NA | NA | NA | NA | NA | NA | Important |
| **Time on positive pressure ventilation support (HF therapy, CPAP, MV)** | | | | | | | | | | |
| 0 | NA | NA | NA | NA | NA | NA | NA | NA | NA | Important |
| Adj HR= adjusted hazard ratio; CI= confidence interval; CPAP= continuous positive airway pressure; HF= high flow; I= Indigenous; MV= mechanical ventilation; NA= not applicable; NIHR= non-Indigenous high risk; NISR= non-Indigenous standard risk; OR= odds ratio (unadjusted).  ^1^ Butler 2019.  ^2^ Serious risk of bias. Downgraded due to concerns about confounding that were sufficient to lower confidence in the estimate of effect. Only ORs from the univariate analysis were reported for this variable.  ^3^ Serious imprecision. Downgraded due to a very small sample size and reasonably wide 95% CIs that include both benefit and harm.  ^4^ Homaira 2016.  ^5^ Serious imprecision. Downgraded due to wide 95% CIs that include reasonable benefit and harm for one of the groups.  ^6^ Prasad 2020.  ^7^ Serious risk of bias. Downgraded due to concerns about the comparability of the cohorts. | | | | | | | | | | |

**Risk factor: timing of illness onset at hospital presentation**

| Quality assessment | | | | | | | No. of patients | Key findings | Quality | Importance |
| --- | --- | --- | --- | --- | --- | --- | --- | --- | --- | --- |
| No. of studies | Design | Risk of bias | Inconsistency | Indirectness | Imprecision | Other considerations |  |  |  |  |
| **Admission to ICU** (aOR for ICU admission) | | | | | | | | | | |
| 1^1^ | Prospective observational (secondary analysis of RCT data) | Not serious | NA | Not serious | Not serious | None | 1,472 | Adj OR 0.78 (95% CI 0.65 to 0.94), *p*=.009. | ⊕⊕⊕⊕  High | Critical |
| **Death** | | | | | | | | | | |
| 0 | NA | NA | NA | NA | NA | NA | NA | NA | NA | Critical |
| **Mechanical ventilation** | | | | | | | | | | |
| 0 | NA | NA | NA | NA | NA | NA | NA | NA | NA | Critical |
| **Admission to hospital** | | | | | | | | | | |
| 0 | NA | NA | NA | NA | NA | NA | NA | NA | NA | Important |
| **Length of stay** | | | | | | | | | | |
| 0 | NA | NA | NA | NA | NA | NA | NA | NA | NA | Important |
| **Time on positive pressure ventilation support (HF therapy, CPAP, MV)** | | | | | | | | | | |
| 0 | NA | NA | NA | NA | NA | NA | NA | NA | NA | Important |
| Adj OR= adjusted odds ratio; CI= confidence interval; CPAP= continuous positive airway pressure; HF= high flow; MV= mechanical ventilation; NA= not applicable.  ^1^ Franklin 2023. In a population of hypoxaemic infants with bronchiolitis. | | | | | | | | | | |
